# Diagnostic Performance of Relative Apical Sparing Across Cardiac Diseases: A Multimodality Systematic Review and Meta-Analysis

**DOI:** 10.3390/jcm15051685

**Published:** 2026-02-24

**Authors:** Andrea Sonaglioni, Giulio Francesco Gramaglia, Gian Luigi Nicolosi, Massimo Baravelli, Michele Lombardo

**Affiliations:** 1 Division of Cardiology, Istituto di Ricovero e Cura a Carattere Scientifico (IRCCS) MultiMedica, 20123 Milan, Italy; massimo.baravelli@multimedica.it (M.B.); michele.lombardo@multimedica.it (M.L.); 2 Department of Emergency, Fondazione Istituto di Ricovero e Cura a Carattere Scientifico (IRCCS) Ca’ Granda, Ospedale Maggiore Policlinico, 20122 Milan, Italy; giulio.gramaglia@unimi.it; 3 Division of Cardiology, Policlinico San Giorgio, 33170 Pordenone, Italy; gianluigi.nicolosi@gmail.com

**Keywords:** relative apical sparing pattern, cardiac amyloidosis, aortic stenosis, hypertrophic cardiomyopathy, hypertensive heart disease, Fabry disease, mitral valve prolapse

## Abstract

**Background:** Relative apical sparing of longitudinal strain is widely used as a diagnostic marker of cardiac amyloidosis. However, similar deformation patterns have been reported in other cardiac diseases, raising concerns regarding disease specificity. A comprehensive multimodality synthesis of the relative apical sparing pattern (RASP) across disease entities is lacking. **Methods:** A systematic review and meta-analysis were conducted according to PRISMA guidelines. PubMed, Scopus, and EMBASE were searched through December 2025 for studies reporting RASP or regional longitudinal strain values allowing standardized RASP calculation. Cardiac amyloidosis and major phenocopies—including aortic stenosis, hypertrophic cardiomyopathy, hypertensive heart disease, Fabry disease, mitral valve prolapse, and other cardiomyopathies—were included. Random-effects models were used to compare cardiac amyloidosis with non-amyloid conditions using standardized mean differences (SMDs), with subgroup analyses according to imaging modality (two-dimensional speckle-tracking echocardiography [2D-STE] versus cardiac magnetic resonance feature tracking [CMR-FT]). **Results:** Fourteen studies (nine 2D-STE and five CMR-FT) were included in the quantitative synthesis. Overall, cardiac amyloidosis was associated with significantly higher RASP compared with non-amyloid conditions (SMD 0.676, 95% CI 0.493–0.860; *p* < 0.001), with substantial heterogeneity (I^2^ = 96.9%). Modality-stratified analyses showed a very large pooled effect for 2D-STE (SMD 2.152, 95% CI 1.354–2.950; I^2^ = 97.6%) and a moderate, homogeneous effect for CMR-FT (SMD 0.594, 95% CI 0.405–0.782; I^2^ = 0%). Sensitivity analyses confirmed robustness. No significant publication bias was detected by Egger’s test. **Conclusions:** Relative apical sparing is not specific to cardiac amyloidosis but is most pronounced in this condition. Its diagnostic magnitude varies across modalities and clinical contexts, supporting a multiparametric, modality-specific interpretation.

## 1. Introduction

Relative apical sparing of longitudinal strain has emerged as one of the most widely recognized imaging signatures of cardiac amyloidosis [1,2]. Since its first description, the preservation of apical deformation in the presence of marked basal and mid-ventricular dysfunction has been increasingly used as a non-invasive marker to support the diagnosis of amyloid cardiomyopathy and to differentiate it from other causes of left ventricular hypertrophy and heart failure [3,4,5].

The relative apical sparing pattern (RASP) is commonly quantified using ratio-based indices derived from regional longitudinal strain, most frequently expressed as the ratio between apical strain and the sum of basal and mid-ventricular strain values [6,7]. This RASP captures the characteristic base-to-apex gradient of myocardial dysfunction observed in cardiac amyloidosis and has demonstrated good diagnostic performance in several single-center and multicenter studies [8,9,10]. As a result, RASP has been incorporated into clinical workflows and is increasingly applied in routine echocardiographic and cardiac magnetic resonance strain analysis.

However, growing evidence suggests that apical sparing is not a pathognomonic feature of cardiac amyloidosis. Similar regional deformation patterns have been reported in other clinical conditions, including pressure overload states such as aortic stenosis, hypertrophic phenotypes such as hypertrophic cardiomyopathy and Fabry disease, mitral valve prolapse (MVP) and selected forms of hypertensive heart disease and advanced cardiomyopathies [11]. These observations raise important clinical questions regarding the specificity of the apical sparing pattern and the magnitude of overlap between cardiac amyloidosis and its phenocopies.

Despite the expanding literature on relative apical sparing, several important gaps remain. First, most available studies focus on binary comparisons between cardiac amyloidosis and a single control group, limiting the ability to contextualize RASP values across a broad spectrum of cardiac diseases. Second, reported RASP values vary substantially across studies, imaging platforms, and post-processing software, making it difficult to establish reference ranges and disease-specific distributions. Third, although multiple meta-analyses have addressed global longitudinal strain in amyloidosis [12,13], a comprehensive quantitative synthesis specifically focused on the RASP across imaging modalities is currently lacking.

Furthermore, with the increasing adoption of cardiac magnetic resonance feature tracking (CMR-FT) as an alternative to speckle-tracking echocardiography (STE) [14], the comparative performance of RASP derived from different imaging techniques has not been systematically evaluated. Whether the magnitude of apical sparing differs between echocardiographic and CMR-based strain analysis and how this affects diagnostic discrimination remains incompletely understood.

Therefore, the aims of the present study were twofold. First, we performed a systematic review of all available studies reporting the RASP across cardiac amyloidosis and other disease entities associated with apical sparing patterns, including aortic stenosis, hypertrophic cardiomyopathy, hypertensive heart disease, Fabry disease, MVP and other cardiomyopathies. Second, we conducted a meta-analysis comparing cardiac amyloidosis with non-amyloid conditions to quantitatively assess the magnitude of RASP differences and to evaluate potential modality-specific effects between STE and CMR-FT.

By integrating descriptive pooled data with quantitative meta-analytic synthesis, this study aims to clarify the clinical meaning of relative apical sparing, to better define its disease specificity, and to provide a comprehensive framework for the interpretation of RASP in daily clinical practice.

## 2. Materials and Methods

This systematic review and meta-analysis were performed in accordance with the Preferred Reporting Items for Systematic Reviews and Meta-Analyses (PRISMA) statement [15]. The PRISMA checklist is provided in the Supplementary Materials (Supplementary Materials S1).

The review protocol was prospectively registered in the INPLASY registry prior to data extraction and statistical analysis (registration number: INPLASY202610080; registration date: 23 January 2026; Supplementary Materials S2).

### 2.1. Search Strategy

A comprehensive literature search was independently conducted by two investigators to identify all relevant studies reporting the RASP or equivalent regional strain-based indices in patients with cardiac amyloidosis and comparator populations.

The electronic databases PubMed, Scopus, and EMBASE were systematically searched from database inception to December 2025. The search strategy combined controlled vocabulary terms and free-text keywords related to cardiac amyloidosis, myocardial strain analysis, and apical sparing patterns.

The following search terms and Boolean operators were used in various combinations:

“cardiac amyloidosis” OR “amyloid cardiomyopathy” OR “ATTR amyloidosis” OR “AL amyloidosis” OR “aortic stenosis” OR “hypertrophic cardiomyopathy” OR “hypertensive heart disease” OR “Fabry disease” OR “mitral valve prolapse” OR “non-amyloid cardiomyopathies” AND

“apical sparing” OR “relative apical sparing pattern” OR “RASP” OR “regional longitudinal strain” AND

“echocardiography” OR “speckle tracking” OR “strain imaging” OR “cardiac magnetic resonance” OR “feature tracking” OR “CMR strain”.

No restrictions were applied regarding language, publication year, or study design. In addition, the reference lists of all included articles and relevant review papers were manually screened to identify potentially eligible studies not captured by the electronic search.

Any discrepancies between reviewers during the screening and selection process were resolved by discussion and consensus. When agreement could not be reached, a third investigator was consulted for adjudication.

### 2.2. Eligibility Criteria

Studies were considered eligible for inclusion if they had an observational design, including cross-sectional, case–control, or cohort studies, and evaluated patients with cardiac amyloidosis and/or other disease entities associated with relative apical sparing patterns, including aortic stenosis, hypertrophic cardiomyopathy, hypertensive heart disease, Fabry disease, MVP and other cardiomyopathies. Eligible studies were required to provide a quantitative assessment of myocardial deformation with reporting of regional longitudinal strain values or direct calculation of the RASP, obtained using STE or CMR-FT. Furthermore, studies had to report clearly defined diagnostic criteria for cardiac amyloidosis and comparator conditions based on histology, imaging findings, or guideline-recommended clinical algorithms, and to provide extractable quantitative data on RASP or on apical, mid-ventricular, and basal longitudinal strain values allowing standardized computation of the RASP, reported as mean ± standard deviation, median with interquartile range, or in a format suitable for statistical transformation. Preference was given to studies reporting baseline clinical and echocardiographic variables, including demographic characteristics, comorbidities, and pharmacological therapy.

Studies were excluded if they did not include strain-based regional deformation analysis or if insufficient information was available to derive the relative apical sparing pattern, if they enrolled mixed or poorly characterized populations without a clear distinction between cardiac amyloidosis and comparator disease groups, if they used experimental or non-clinical imaging protocols without standardized strain quantification, if quantitative data were insufficient to allow for effect size calculation or pooled analysis, or if they were non-original publications such as editorials, conference abstracts, letters, case reports, narrative reviews, expert opinions, or guidelines.

### 2.3. Study Selection and Data Extraction

Two investigators independently screened all retrieved records by title and abstract, followed by full-text evaluation of potentially eligible studies according to the predefined inclusion and exclusion criteria. Disagreements regarding study eligibility were resolved by discussion and consensus, and when consensus could not be achieved, a third reviewer was consulted for adjudication. Data extraction was independently performed by the same investigators using a standardized and pre-specified data collection form. Extracted variables included study characteristics such as first author, year of publication, country, study design, imaging modality, and sample size for each study group; demographic and anthropometric parameters including age, sex distribution, body mass index, and body surface area; clinical comorbidities and cardiovascular risk factors such as hypertension, diabetes mellitus, dyslipidemia, smoking history, atrial fibrillation, and history of coronary artery disease; laboratory parameters when available, including hemoglobin, renal function indices, natriuretic peptides, and serum biomarkers; echocardiographic and structural parameters including left ventricular wall thickness, relative wall thickness, left ventricular mass index (LVMi), chamber dimensions, left ventricular ejection fraction, stroke volume index, indices of diastolic function, left atrial volume index, pulmonary artery systolic pressure, right ventricular systolic function parameters, and presence of pericardial effusion; strain-derived parameters including global longitudinal strain, mean basal, mid-ventricular, and apical longitudinal strain values (calculated as the average of segmental values within each ventricular level), directly reported RASP values [defined as the ratio between mean apical longitudinal strain and the sum of mean basal and mid-ventricular longitudinal strain values: RASP = |apical LS|/(|basal LS| + |mid-ventricular LS|)], and study-specific diagnostic cut-offs when available. When only regional longitudinal strain values were reported, RASP was recalculated using this standardized formula. Studies adopting alternative formulations (e.g., apical/basal strain ratios or different denominators) were harmonized to the predefined definition when sufficient data were available; otherwise, the originally reported RASP metric was retained and recorded accordingly; baseline pharmacological therapy including antiplatelet agents, anticoagulants, beta-blockers, angiotensin-converting enzyme inhibitors, angiotensin receptor blockers, calcium channel blockers, diuretics, statins, antiarrhythmic drugs, and disease-specific therapies; and summary statistics including means with standard deviations or medians with interquartile ranges, together with corresponding confidence intervals and *p*-values. When numerical data were reported exclusively in graphical format, values were extracted using WebPlotDigitizer (version 4.6), and when necessary for quantitative synthesis, medians and interquartile ranges were converted into means and standard deviations using established statistical conversion methods. All extracted data were systematically cross-checked for accuracy, and discrepancies between reviewers were resolved by re-evaluation of the original articles until agreement was reached.

### 2.4. Methodological Quality Appraisal and Bias Assessment

The methodological quality and risk of bias of the included studies were independently evaluated by two investigators using the National Institutes of Health (NIH) Quality Assessment Tool for Observational Cohort and Cross-Sectional Studies [16]. This tool assesses multiple methodological domains related to study design, population selection, case and control definition, exposure assessment, outcome ascertainment, blinding procedures, and control of confounding factors. Each study was evaluated across all predefined criteria and classified according to NIH guidance.

Inter-rater agreement between reviewers was quantified using Cohen’s kappa coefficient. Any disagreement regarding individual domain ratings or overall quality classification was resolved through discussion and re-evaluation of the original manuscripts until consensus was achieved.

### 2.5. Statistical Analysis

For quantitative synthesis, the primary meta-analysis compared cardiac amyloidosis groups versus non-amyloid conditions, including all alternative disease entities associated with relative apical sparing patterns such as aortic stenosis, hypertrophic cardiomyopathy, hypertensive heart disease, Fabry disease, MVP and other cardiomyopathies, and healthy controls. To ensure statistical independence of effect size estimates, when a single study reported multiple non-amyloid conditions sharing the same cardiac amyloidosis cohort, the comparator arms were combined into a single pooled non-amyloid group using standard methods for pooling means and standard deviations. This approach yielded one independent comparison per imaging modality per study and avoided double-counting of the cardiac amyloidosis cohort.

Comparative meta-analyses were performed using random-effects models. Effect sizes for continuous outcomes were expressed as standardized mean differences (SMDs) with corresponding 95% confidence intervals, while odds ratios were calculated for categorical variables when applicable. When studies reported continuous data as medians with interquartile ranges or ranges, means and standard deviations were estimated using validated statistical conversion methods. These transformed values were used exclusively for meta-analytic pooling and were not included in descriptive summaries.

A random-effects model based on the DerSimonian–Laird method was selected a priori to account for expected clinical and methodological heterogeneity across studies. Between-study heterogeneity was quantified using the I^2^ statistic, with values of approximately 25%, 50%, and 75% interpreted as low, moderate, and high heterogeneity.

Pre-specified subgroup analyses were performed according to imaging modality to evaluate potential differences in effect size estimates between studies using two-dimensional (2D)-STE and those using CMR-FT. Separate random-effects models were constructed for each imaging subgroup, and pooled SMDs with corresponding 95% confidence intervals were calculated. Between-subgroup differences were formally assessed using the Q-test for subgroup differences.

Publication bias and small-study effects were evaluated through visual inspection of funnel plots and formally tested using Egger’s regression asymmetry test.

Meta-regression analyses were undertaken to investigate potential sources of between-study heterogeneity in effect size estimates. These analyses were conducted on the overall dataset using mixed-effects models within a random-effects framework. Given the limited number of included studies, each covariate was evaluated separately in univariable (one-covariate-at-a-time) models to minimize risks of overfitting and collinearity. The number of studies contributing to each model varied according to data availability for the respective covariate, and no imputation of missing study-level data was performed. The intercept term was retained for model specification but was not interpreted clinically. Residual between-study variance (τ^2^) was examined after inclusion of each covariate to assess whether the moderator reduced unexplained heterogeneity. Owing to the small number of studies, meta-regression findings were considered exploratory and interpreted cautiously.

Two complementary sensitivity strategies were applied. First, to evaluate the robustness of pooled estimates to the choice of variance estimator and inferential method, additional random-effects analyses were conducted using restricted maximum likelihood (REML) estimation for τ^2^, with Hartung–Knapp–Sidik–Jonkman adjustment for confidence intervals. Ninety-five percent prediction intervals were also calculated to estimate the range of true effects that might be expected in future comparable settings. Second, an influence analysis using a leave-one-out approach was performed within the heterogeneous 2D-STE subgroup to assess the impact of individual studies on pooled estimates.

All statistical analyses were performed using Comprehensive Meta-Analysis software (version 3.0; Biostat, Englewood, NJ, USA) and IBM SPSS Statistics (version 29.0; IBM Corp., Armonk, NY, USA). All tests were two-tailed, with *p* < 0.05 considered statistically significant.

## 3. Results

### 3.1. Study Identification and Selection Process

**Figure 1:**
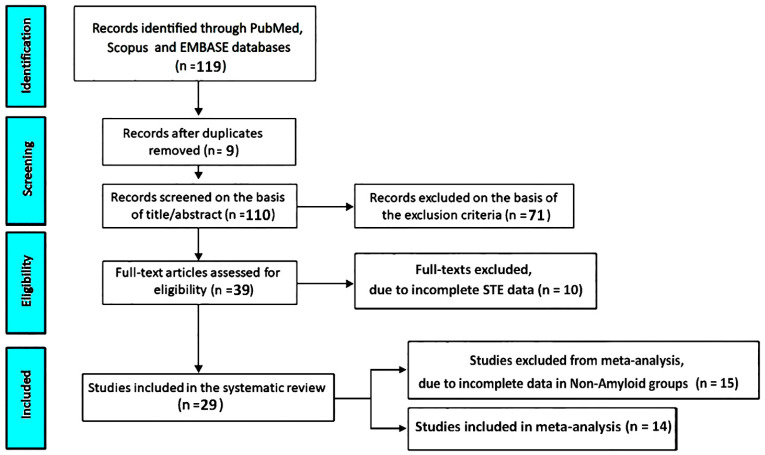
Flow diagram illustrating the literature screening and study selection procedure following PRISMA guidelines. STE, speckle tracking echocardiography.

The systematic literature search initially retrieved 119 records. After removal of 9 duplicate entries (7.6%), 110 unique articles remained for screening. Following title and abstract evaluation, 71 studies (59.7%) were excluded according to the predefined eligibility criteria. Consequently, 39 full-text articles (32.8%) were assessed for eligibility. Among these, 10 studies (8.4%) were excluded due to incomplete STE data. Ultimately, 29 studies (24.4%) [17,18,19,20,21,22,23,24,25,26,27,28,29,30,31,32,33,34,35,36,37,38,39,40,41,42,43,44,45] reporting RASP measurements were included in the qualitative systematic review, and 14 of these (11.8%) [17,19,20,21,30,32,33,35,36,40,41,42,43,44] provided suitable data for inclusion in the quantitative meta-analysis comparing cardiac amyloidosis with non-amyloid conditions using speckle-tracking–derived RASP values (Figure 1).

### 3.2. Clinical and Methodological Characteristics of Included Studies

The studies included in the present analysis were published between 2015 and 2025 and were conducted across multiple geographic regions, including Europe, Asia, North America, and Oceania. Specifically, contributing investigations originated from countries such as South Korea, Denmark, the United States, Australia, China, Japan, Italy, France, Germany, Portugal, Hungary, Canada, and the Netherlands, reflecting a broad international representation of patient populations.

Most studies adopted a retrospective observational design, although several prospective cohorts were also included. The majority were monocentric investigations, with a smaller proportion conducted across multiple centers. Sample sizes varied substantially, ranging from small exploratory cohorts with fewer than 20 participants to large observational populations exceeding 500 subjects, particularly in studies focusing on senile amyloidosis or large registry-based cohorts.

Regarding imaging methodology, relative apical sparing was predominantly assessed using 2D-STE, which represented the most frequently employed technique. A smaller number of studies used CMR-FT, three-dimensional STE, or FT computed tomography. Several commercial software platforms were utilized for strain analysis, including GE EchoPAC, Philips QLAB, Siemens systems, TomTec, Medis Medical Imaging, Circle Cardiovascular Imaging, and MyoStrain, contributing to methodological heterogeneity across studies.

The included populations encompassed a wide spectrum of cardiac phenotypes. Cardiac amyloidosis cohorts were commonly compared with patients affected by hypertensive heart disease, hypertrophic cardiomyopathy, Fabry disease, aortic stenosis, dilated cardiomyopathy, end-stage renal disease, MVP, and healthy subjects. Overall, patients with cardiac amyloidosis were generally older and more frequently male than most comparator populations.

Across studies reporting amyloidosis subtypes, immunoglobulin light-chain (AL) cardiac amyloidosis accounted for a median of 51.3% (IQR 24.0–93.5) of cases, whereas transthyretin (ATTR) cardiac amyloidosis represented 48.7% (IQR 29.0–76.2). Wild-type transthyretin amyloidosis (ATTRwt) was reported less frequently, with a median prevalence of 2.0% (IQR 2.0–4.2) among studies providing this information. Undetermined amyloidosis subtypes were uncommon and reported in a limited number of cohorts (median 2.0%, IQR 2.0–16.7).

Biopsy-proven cardiac amyloidosis was reported in a median of 100% (IQR 44.2–100) of patients among studies providing histological confirmation. Non-invasive diagnosis using technetium-99m pyrophosphate (99mTc-PYP) scintigraphy or CMR was available in a median of 58.0% (IQR 35.9–88.9) of patients.

Reported mean RASP values demonstrated substantial variability across disease groups and imaging modalities. Cardiac amyloidosis cohorts consistently exhibited higher RASP values compared with non-amyloid populations, with mean values ranging approximately from 0.6 to over 2.0 depending on study design, imaging technique, and disease severity. In contrast, comparator groups such as hypertensive heart disease, hypertrophic cardiomyopathy, Fabry disease, and healthy controls typically showed lower or intermediate RASP values. Despite heterogeneity in study protocols and population characteristics, all included investigations provided comparative quantitative data on relative apical sparing across different cardiac phenotypes, forming the basis for the pooled descriptive analysis and subsequent meta-analytic synthesis (Table 1).

### 3.3. Systematic Review Findings and Pooled Characteristics Across Disease Groups

The systematic review included studies evaluating relative apical sparing patterns across cardiac amyloidosis, aortic stenosis, hypertrophic cardiomyopathy, hypertensive heart disease, Fabry disease, other cardiomyopathy phenotypes, MVP and healthy controls. Pooled study-level characteristics and echocardiographic parameters are summarized in Table 2.

Descriptive pooled summaries suggested differences across disease phenotypes with respect to cardiac chamber geometry, ventricular remodeling, and functional impairment. Cardiac amyloidosis was characterized by relatively small left ventricular cavity dimensions despite increased wall thickness and mass, with lower left ventricular end-diastolic diameter and indexed end-diastolic volume compared with pressure-overload states such as aortic stenosis and hypertensive heart disease, and smaller cavity sizes than dilated or mixed cardiomyopathy phenotypes. This pattern is consistent with the typical restrictive-infiltrative remodeling of amyloid cardiomyopathy, in which concentric wall thickening occurs in the absence of true chamber dilation. In contrast, aortic stenosis and hypertensive heart disease tended to show moderately enlarged ventricular cavities compatible with adaptive remodeling to chronic pressure overload, while hypertrophic cardiomyopathy showed intermediate cavity dimensions associated with asymmetric or concentric hypertrophy. Healthy controls generally exhibited larger relative cavity dimensions when indexed to body surface area, reflecting preserved physiological geometry. Left atrial volumes appeared higher in cardiac amyloidosis, consistent with chronically elevated filling pressures and restrictive physiology, and numerically exceeded those observed in several other disease groups.

Although left ventricular ejection fraction appeared moderately preserved across several disease groups, cardiac amyloidosis showed lower pooled ejection fraction values compared with hypertrophic cardiomyopathy, Fabry disease, and healthy controls in descriptive summaries, suggesting early systolic impairment in advanced infiltrative disease. This reduction was less marked than in overt dilated cardiomyopathy phenotypes but remained clinically relevant in the context of restrictive ventricular mechanics. Global longitudinal strain values indicated more pronounced impairment of longitudinal deformation in amyloidosis relative to other groups at the descriptive level, consistent with preferential involvement of subendocardial longitudinal fibers despite relatively preserved ejection fraction. Pressure-overload and hypertrophic phenotypes demonstrated intermediate strain impairment, whereas Fabry disease and healthy subjects showed more preserved strain values.

Diastolic indices in cardiac amyloidosis were characterized by higher E/A ratios and elevated E/e’ values in pooled descriptive analyses, reflecting restrictive filling physiology and increased filling pressures. In contrast, aortic stenosis and hypertensive heart disease showed patterns compatible with impaired relaxation and pseudonormal filling, while hypertrophic cardiomyopathy demonstrated intermediate abnormalities and Fabry disease and healthy cohorts exhibited comparatively preserved diastolic parameters.

**Figure 2:**
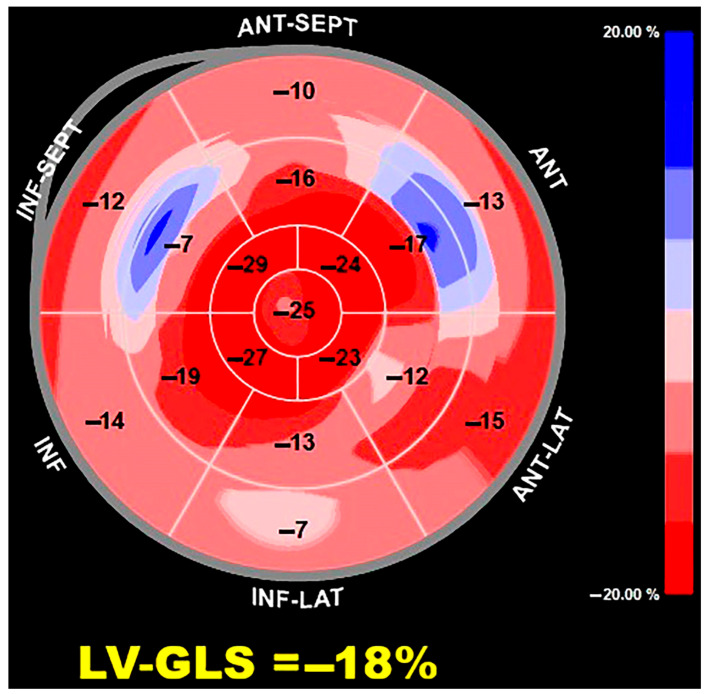
Representative bull’s-eye (polar) map illustrating the base-to-apex gradient of longitudinal strain in a patient with cardiac amyloidosis. The map demonstrates relatively preserved apical strain compared with reduced basal and mid-ventricular strain, consistent with the typical “apical sparing” pattern. Global longitudinal strain and segmental strain values are displayed.

Finally, cardiac amyloidosis demonstrated a distinct base-to-apex deformation gradient in pooled summaries, characterized by reduced basal and mid-ventricular longitudinal strain with relative preservation of apical strain, resulting in higher RASP values compared with other disease categories. Although intermediate apical sparing patterns were also observed in aortic stenosis, hypertrophic cardiomyopathy, Fabry disease, and other cardiomyopathy phenotypes, amyloidosis appeared to show the most pronounced regional strain heterogeneity at the descriptive level, consistent with its characteristic infiltrative myocardial distribution. Healthy individuals and hypertensive heart disease cohorts generally exhibited lower relative apical sparing values, reflecting more homogeneous myocardial deformation patterns. Figure 2 shows a representative example of the base-to-apex gradient in longitudinal strain deformation detected in a patient with cardiac amyloidosis.

### 3.4. Meta-Analysis Results: Cardiac Amyloidosis Versus Non-Amyloid Conditions

Fourteen studies were included in the meta-analysis, comprising nine echocardiography-based investigations using 2D-STE and five studies employing CMR-FT.

**Figure 3:**
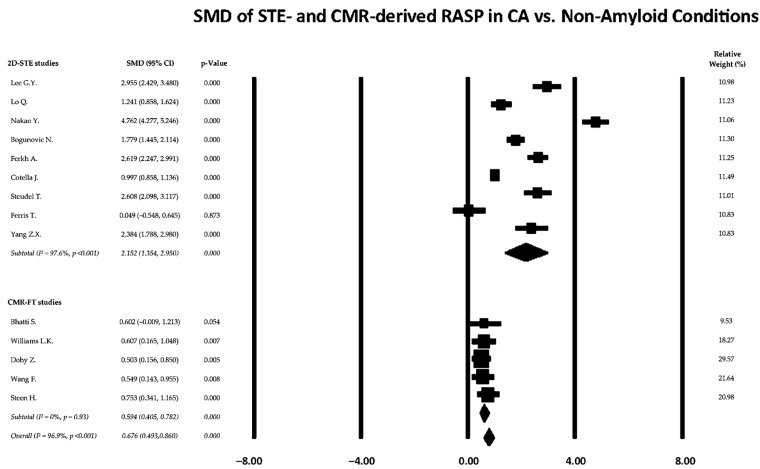
Forest plot of standardized mean differences (SMDs) for relative apical sparing comparing cardiac amyloidosis and non-amyloid conditions. Results are stratified by imaging modality, including two-dimensional speckle-tracking echocardiography (2D-STE) and cardiac magnetic resonance feature tracking (CMR-FT). Squares represent individual study estimates, with size proportional to study weight, and horizontal lines indicate 95% confidence intervals. Diamonds denote pooled random-effects estimates for each subgroup and for the overall analysis. Between-study heterogeneity was quantified using the I^2^ statistic [17,19,20,21,30,32,33,35,36,40,41,42,43,44].

Figure 3 presents the forest plot of pooled SMDs comparing RASP values between patients with cardiac amyloidosis and non-amyloid control groups, with results stratified according to imaging modality.

Across all imaging modalities and comparator groups, cardiac amyloidosis was associated with higher RASP values compared with non-amyloid conditions. The pooled random-effects analysis demonstrated a standardized mean difference of 0.676 (95% CI 0.493–0.860; *p* < 0.001), indicating a moderate effect size favoring cardiac amyloidosis. Between-study heterogeneity was extremely high (I^2^ = 96.9%; *p* < 0.001), reflecting substantial variability in effect magnitude across studies.

**Figure 4:**
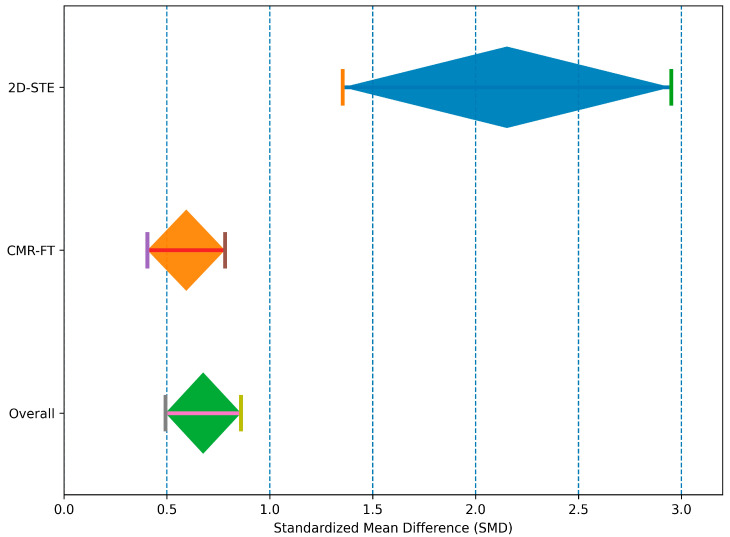
Pooled standardized mean differences for relative apical sparing pattern comparing cardiac amyloidosis and non-amyloid conditions, stratified by imaging modality. Diamonds represent random-effects estimates, and horizontal lines indicate 95% confidence intervals.

When stratified by imaging modality (Figure 4), important differences emerged. In the 2D-STE subgroup (nine studies), the pooled SMD was 2.152 (95% CI 1.354–2.950; *p* < 0.001), indicating a very large effect size, but with extremely high heterogeneity (I^2^ = 97.6%, *p* < 0.001), suggesting marked variability in study populations and methodological characteristics. In contrast, the CMR-FT subgroup (five studies) showed a pooled SMD of 0.594 (95% CI 0.405–0.782; *p* < 0.001), consistent with a moderate effect size, and no evidence of heterogeneity (I^2^ = 0%, *p* = 0.93), indicating high consistency across studies. These findings suggest that the overall heterogeneity of the meta-analysis is primarily driven by the 2D-STE studies, whereas CMR-FT results appear more homogeneous and methodologically consistent.

### 3.5. Comparative Characteristics of STE and CMR-FT Study Cohorts

Table 3 presents a structured comparison of study-level characteristics between investigations using 2D-STE and those employing CMR-FT.

Baseline clinical and echocardiographic variables of the cardiac amyloidosis cohorts were summarized as medians with interquartile ranges to account for distributional variability and the limited number of studies per subgroup.

Across imaging modalities, cardiac amyloidosis cohorts showed broadly comparable distributions in age, body surface area, systolic blood pressure, heart rate, left ventricular ejection fraction, and E/e’ ratio. The proportion of male patients and the prevalence of diabetes were also similar between STE- and CMR-based investigations.

A difference was observed in LVMI, which demonstrated higher median values and a wider interquartile range among studies using 2D-STE compared with those employing CMR-FT. In contrast, CMR-FT studies showed lower and more narrowly distributed LVMI values at the study level.

The table additionally details the composition of comparator populations included in each modality subgroup. Healthy control cohorts were incorporated in a subset of investigations within both modalities, although their representation differed numerically between STE and CMR-FT studies. Hypertensive heart disease comparators were present in both subgroups, whereas hypertrophic cardiomyopathy was included predominantly in CMR-FT investigations.

Finally, the distribution of strain analysis software platforms differed between subgroups. Most STE investigations were conducted using GE-based systems, whereas CMR-FT studies were performed exclusively with non-GE software platforms.

### 3.6. Assessment of Small-Study Effects and Publication Bias

**Figure 5:**
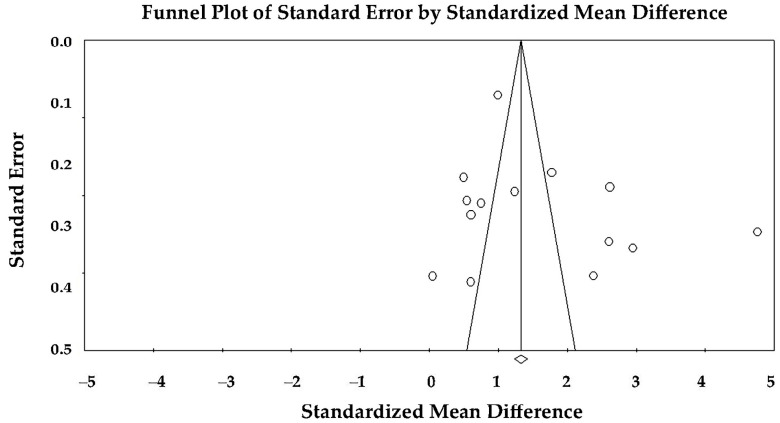
Assessment of publication bias for the association between cardiac amyloidosis and relative apical sparing using funnel plot analysis.

Visual inspection of the funnel plot did not suggest substantial asymmetry. Egger’s regression test did not demonstrate statistically significant evidence of small-study effects (two-tailed *p* = 0.237), indicating no significant evidence of publication bias among the studies included in the meta-analysis (Figure 5).

### 3.7. Results of Meta-Regression and Sensitivity Analyses

Meta-regression analyses were conducted on the overall dataset (14 studies) to explore whether demographic, clinical, echocardiographic, and methodological characteristics influenced the association between relative apical sparing pattern and cardiac amyloidosis. No significant moderating effects were observed for any of the evaluated covariates (all *p* > 0.05; Table 4). Specifically, age, sex distribution, cardiovascular risk factors, body surface area, systolic blood pressure, heart rate, LVMi, left ventricular ejection fraction, diastolic function parameters (E/e’), amyloid subtype (AL-cardiac amyloidosis), and imaging software platform were not significantly associated with variability in effect size estimates.

Given the absence of statistical heterogeneity within the CMR-FT subgroup (I^2^ = 0%), subgroup-specific meta-regression was not performed for CMR-based studies. However, the overall meta-regression findings should be interpreted with caution due to the relatively small number of included studies, which may limit statistical power to detect moderate moderator effects.

These findings suggest that the association between relative apical sparing pattern and cardiac amyloidosis appears broadly consistent across study-level characteristics, although residual heterogeneity remains largely unexplained.

To evaluate the robustness and stability of the primary findings, sensitivity analyses were performed using complementary statistical approaches. Using a random-effects model with REML estimation, the pooled SMD was 1.59 (95% CI 0.91–2.27; *p* < 0.001), indicating a moderate-to-large effect size favoring cardiac amyloidosis. Application of the Hartung–Knapp–Sidik–Jonkman adjustment yielded directionally consistent results with modestly wider confidence intervals, confirming the robustness of the overall association to alternative inferential approaches. Between-study heterogeneity remained extremely high (I^2^ ≈ 96.8%). Consistent with this variability, the 95% prediction interval ranged from −1.30 to 4.47, indicating that the magnitude of the true effect may vary substantially across different clinical and methodological settings.

A second sensitivity analysis was conducted using a leave-one-out influence approach within the 2D-STE subgroup, given the substantial heterogeneity observed in this subset (I^2^ = 97.6%). Sequential exclusion of individual studies did not materially alter the direction of the association, and cardiac amyloidosis consistently demonstrated higher RASP values compared with non-amyloid conditions across all iterations. The pooled SMD remained statistically significant in every model, ranging from 2.09 (95% CI 0.41–3.77) to 2.98 (95% CI 0.87–5.10). Although the magnitude of the effect varied across iterations, the overall effect remained large.

Collectively, these findings indicate that the observed association was not driven by any single 2D-STE study. Leave-one-out analyses were not performed for CMR-FT studies due to the absence of between-study heterogeneity (I^2^ = 0%), reflecting high consistency of effect estimates within that subgroup.

### 3.8. Risk of Bias Assessment

Inter-reviewer agreement for the risk-of-bias assessment was high, with substantial reliability (Cohen’s κ = 0.82). Using the NIH Quality Assessment Tool for Observational Cohort and Cross-Sectional Studies [16], the methodological quality of included investigations was judged as predominantly fair to good, with most studies fulfilling the majority of predefined criteria (Supplementary Materials S3).

Across studies, research objectives were clearly stated, and study populations were adequately defined and characterized. Eligibility criteria were generally well described, and imaging acquisition protocols were largely standardized. Consistent operational definitions of relative apical sparing were applied in most investigations. However, formal sample size justification was infrequently reported, blinding of outcome assessors was inconsistently described, and adjustment for potential confounding variables was incomplete in a subset of studies.

Although these methodological limitations may affect internal validity, they are unlikely to have introduced systematic bias in the direction of the pooled effect estimates.

A visual summary of the domain-level risk-of-bias assessment is provided in Supplementary Materials S4 (traffic-light plot) [17,19,20,21,30,32,33,35,36,40,41,42,43,44], and the aggregated distribution of “YES”, “NO”, and “NR” judgments across domains is presented in Supplementary Materials S5 [17,19,20,21,30,32,33,35,36,40,41,42,43,44].

## 4. Discussion

### 4.1. Main Findings

This study provides the first comprehensive integration of systematic descriptive evidence and quantitative meta-analytic synthesis specifically focused on relative apical sparing pattern across cardiac amyloidosis and alternative disease entities associated with apical sparing patterns. Several key findings emerge.

First, pooled evidence from the systematic review confirms that relative apical sparing is not exclusive to cardiac amyloidosis but is observed across multiple pathological conditions, including aortic stenosis, hypertrophic cardiomyopathy, Fabry disease, hypertensive heart disease, MVP, and other cardiomyopathic phenotypes. Despite this overlap, cardiac amyloidosis consistently demonstrated higher absolute RASP values and a more pronounced base-to-apex deformation gradient. This pattern occurred within a characteristic restrictive-infiltrative phenotype marked by increased wall thickness and mass, relatively small ventricular cavity dimensions, left atrial enlargement, impaired longitudinal systolic function, and advanced diastolic dysfunction. The convergence of concentric remodeling, reduced ventricular compliance, and elevated filling pressures characterizes the restrictive phenotype of amyloid cardiomyopathy. Preferential involvement of basal longitudinal fibers likely contributes to the distinctive strain distribution observed in this condition.

Second, the quantitative meta-analysis confirmed that cardiac amyloidosis is associated with significantly higher RASP values compared with non-amyloid conditions, with a pooled standardized mean difference in the moderate-to-large range. Importantly, the analytic framework was refined to ensure statistical independence by combining multiple comparator arms within individual studies into a single pooled non-amyloid group when a shared amyloidosis cohort was present. Re-analysis using this approach yielded directionally consistent effect size estimates, reinforcing the robustness of the association while providing more conservative precision estimates. However, between-study heterogeneity was extremely high, and prediction interval analyses demonstrated a wide range of plausible true effects across different study settings, underscoring that the magnitude of the association is highly context-dependent rather than uniform across populations.

Meta-regression analyses performed on the overall dataset did not identify demographic, clinical, echocardiographic, or methodological covariates that significantly modified the association between relative apical sparing pattern and cardiac amyloidosis. However, given the limited number of included studies, these results should be interpreted cautiously. The persistence of residual heterogeneity despite adjustment for available study-level covariates suggests that unmeasured biological and technical factors—such as amyloid subtype distribution, disease stage, myocardial infiltration burden, acquisition protocols, and vendor-specific strain algorithms—may contribute to variability in reported RASP values. This residual variability reinforces the probabilistic nature of relative apical sparing pattern as a diagnostic marker rather than a fixed quantitative discriminator.

Third, modality-stratified analyses revealed differences in pooled effect sizes between studies using 2D-STE and those employing CMR-FT. Although STE studies demonstrated numerically larger pooled effects, interpretation of this finding requires caution. Study-level comparison between modality subgroups showed differences in LVMi distribution, comparator composition (notably the higher representation of hypertrophic cardiomyopathy in CMR-FT studies), and strain software platforms. These factors indicate that the observed subgroup differences likely reflect a combination of technical modality-related characteristics and underlying differences in study populations and methodological approaches, rather than a purely modality-driven effect. Consequently, diagnostic thresholds derived from one imaging modality should not be assumed to be directly transferable to another without validation. Taken together, these findings highlight that both biological heterogeneity and methodological diversity contribute to variability in reported effect sizes.

Fourth, comparator-specific analyses demonstrated that the discriminatory performance of RASP varies according to the reference population. Larger effect sizes were observed when cardiac amyloidosis was compared with hypertensive heart disease, Fabry disease, and healthy controls, whereas smaller effect sizes were seen in comparisons with hypertrophic cardiomyopathy and advanced cardiomyopathic phenotypes. These findings underscore that the specificity of apical sparing is context-dependent and influenced by the remodeling pattern of the comparator condition.

Finally, the stability of the overall findings was supported by leave-one-out sensitivity analyses restricted to the heterogeneous STE subgroup, which demonstrated consistent direction and magnitude of effect. Publication bias assessment using funnel plot inspection and Egger’s regression testing did not reveal significant small-study effects. Collectively, these results indicate that relative apical sparing pattern represents a reproducible and quantitatively significant feature of cardiac amyloidosis; however, it should be interpreted within a broader clinical and imaging framework rather than as an isolated pathognomonic marker.

### 4.2. Pathophysiological Interpretation of Relative Apical Sparing Across Disease Entities

The heterogeneous distribution of myocardial deformation underlying the relative apical sparing pattern likely reflects disease-specific mechanisms of myocardial involvement and remodeling. In cardiac amyloidosis, the preferential impairment of basal and mid-ventricular segments has been attributed to the regional distribution of amyloid deposition, microvascular dysfunction, and transmural infiltration gradients [46,47,48]. Histopathological and imaging studies have demonstrated higher amyloid burden and interstitial expansion in basal segments, leading to disproportionate impairment of longitudinal fiber shortening and preservation of apical mechanics in earlier and intermediate disease stages [49,50,51,52]. This characteristic base-to-apex gradient explains the consistently higher RASP values observed in amyloid cardiomyopathy across imaging modalities.

In contrast, in pressure-overload conditions such as aortic stenosis, apical sparing appears to arise primarily from concentric hypertrophic remodeling and differential wall stress distribution rather than infiltrative pathology. Basal segments are exposed to higher systolic wall stress and mechanical load, which may preferentially impair basal longitudinal function while relatively preserving apical deformation [53,54]. This mechanism is consistent with the intermediate RASP values observed in aortic stenosis cohorts and with the partial overlap between pressure-overload phenotypes and amyloid-related deformation patterns.

Hypertrophic cardiomyopathy and Fabry disease represent additional phenocopies in which regional strain heterogeneity can mimic apical sparing. In hypertrophic cardiomyopathy, asymmetric hypertrophy, fiber disarray, and segmental fibrosis contribute to heterogeneous longitudinal strain patterns, often involving basal septal segments [55]. Similarly, in Fabry disease, glycosphingolipid accumulation and focal myocardial fibrosis predominantly affect inferolateral and basal regions, generating deformation gradients that may resemble those observed in infiltrative cardiomyopathies [56]. These pathophysiological features are consistent with the intermediate RASP values identified in these populations and support the concept that apical sparing reflects a shared mechanical phenotype rather than a disease-specific signature.

Importantly, the relative preservation of apical strain observed across multiple disease entities suggests that apical myocardial segments may be intrinsically more resistant to early mechanical dysfunction, potentially due to differences in myocardial fiber architecture, perfusion patterns, and regional wall stress distribution. However, the magnitude of the base-to-apex gradient appears to be greatest in cardiac amyloidosis, which may explain the superior discriminatory performance of RASP in this population despite incomplete disease specificity [57].

Taken together, these observations indicate that relative apical sparing pattern should be interpreted as a marker of regional myocardial vulnerability rather than as a pathognomonic sign of amyloid infiltration. Integrating RASP with clinical context, morphological features, and complementary imaging findings is therefore essential to improve diagnostic accuracy and avoid misclassification in patients with hypertrophic or infiltrative phenotypes.

Beyond regional longitudinal strain gradients, 2D-STE has increasingly expanded its role in the comprehensive phenotyping of cardiomyopathies. In particular, atrial strain analysis has emerged as a sensitive marker of early diastolic dysfunction and atrial myopathy, providing incremental diagnostic and prognostic information in infiltrative and hypertrophic conditions [58]. In cardiac amyloidosis, impaired left atrial reservoir and conduit strain may precede overt systolic dysfunction and reflect atrial infiltration, stiffness, and elevated filling pressures. Similarly, in hypertrophic and pressure-overload phenotypes, atrial strain abnormalities may capture chronic hemodynamic burden and remodeling beyond what is detectable by conventional volumetric parameters.

In addition, myocardial work indices derived from pressure–strain loop analysis offer a load-adjusted assessment of myocardial performance, potentially overcoming some limitations of deformation parameters that are sensitive to afterload conditions. Myocardial work efficiency and constructive work have demonstrated promising utility in differentiating cardiomyopathy phenotypes and in refining risk stratification. The integration of ventricular strain patterns, atrial deformation analysis, and myocardial work metrics may therefore provide a more comprehensive and pathophysiologically grounded characterization of myocardial dysfunction across infiltrative and non-infiltrative cardiomyopathies. Recent investigations have highlighted the incremental value of atrial strain and myocardial work assessment within multiparametric imaging frameworks, supporting their potential role in enhancing diagnostic precision beyond isolated longitudinal strain indices [59,60,61].

### 4.3. Other Causes of Relative Apical Sparing

Beyond infiltrative and hypertrophic cardiomyopathies, several additional conditions have been reported to exhibit relative apical sparing patterns, further supporting the concept that this deformation phenotype is not disease-specific but reflects heterogeneous mechanical and geometric influences on myocardial function. The studies included in the “other forms” category highlight distinct non-infiltrative mechanisms capable of generating a base-to-apex strain gradient, with important clinical implications.

One relevant subgroup includes patients with advanced renal disease and systemic metabolic alterations, in whom myocardial remodeling, microvascular dysfunction, and volume overload may contribute to regional strain heterogeneity [62,63]. In these populations, basal segments appear particularly vulnerable to loading conditions and fibrosis-related stiffness, leading to relative preservation of apical deformation despite the absence of myocardial infiltration. These findings suggest that systemic disease burden and extracardiac comorbidities may modulate deformation patterns independently of classical cardiomyopathy phenotypes.

Additional evidence indicates that non-ischemic dilated cardiomyopathy (NIDCM) may also exhibit a relative apical sparing pattern [23]. In contrast to ischemic cardiomyopathy, where strain reduction is more homogeneous, patients with NIDCM demonstrate disproportionately reduced basal longitudinal strain with relative preservation of apical segments, resulting in a characteristic base-to-apex deformation gradient. This pattern likely reflects diffuse myocardial remodeling, altered fiber architecture, and heterogeneous wall stress distribution that is typical of non-ischemic myocardial disease.

Similarly, left ventricular non-compaction cardiomyopathy (LVNC) has been shown to display altered longitudinal strain gradients resembling relative apical sparing [33]. Despite globally impaired myocardial mechanics, LVNC patients maintain intraventricular baso-apical strain gradients comparable to those observed in cardiac amyloidosis, suggesting that trabecular architecture, abnormal myocardial compaction, and segmental mechanical inefficiency may contribute to regional deformation heterogeneity.

**Figure 6:**
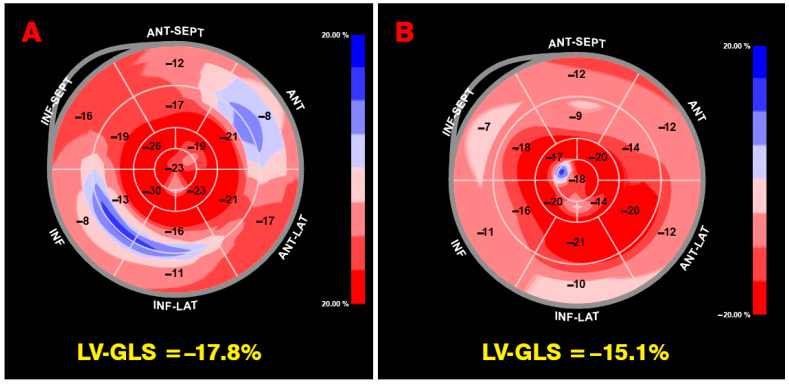
Representative 2D speckle-tracking echocardiography–derived left ventricular global longitudinal strain bull’s-eye maps in a healthy individual with mitral valve prolapse (**A**) and a patient with pectus excavatum (**B**). In both cases, the LV-GLS bull’s-eye maps demonstrate reduced basal segment deformation with relative preservation of apical strain, resulting in an apparent apical sparing pattern. These findings highlight the potential influence of thoracic morphology on regional myocardial deformation patterns independent of myocardial infiltration.

Our study group identified a novel and clinically relevant mechanism demonstrating that thoracic morphology can directly influence regional myocardial mechanics across various clinical settings [64,65]. Specifically, patients with a reduced anterior–posterior chest dimension may exhibit relative apical sparing even in the absence of amyloid infiltration or hypertrophic cardiomyopathy. In such cases, basal left ventricular segments are mechanically constrained by anterior thoracic deformity and diminished retrosternal space, whereas apical segments retain greater freedom of motion. This asymmetric external constraint produces an apparent base-to-apex deformation gradient that closely mimics the classical apical sparing pattern observed in cardiac amyloidosis. Representative examples of STE–derived left ventricular global longitudinal strain bull’s-eye maps obtained in a healthy individual with MVP and in a patient with pectus excavatum are shown in Figure 6.

These findings are further supported by recent evidence in patients with MVP. A large meta-analysis demonstrated that MVP is associated with preferential impairment of basal inferolateral longitudinal strain and relative preservation of apical deformation, resulting in a reproducible apical sparing phenotype [66]. Notably, MVP patients frequently exhibit a narrow anteroposterior thoracic diameter and increased chest wall indices, which may exacerbate basal myocardial constraint and contribute to reduced basal strain values [67]. This reinforces the concept that thoracic geometry represents a key extrinsic modulator of myocardial deformation patterns.

These observations have important diagnostic implications. They indicate that relative apical sparing may arise not only from intrinsic myocardial pathology but also from extrinsic mechanical factors related to chest wall geometry and cardiac positioning. Failure to recognize this phenomenon may lead to false-positive suspicion of amyloidosis, particularly in patients with slender body habitus, chest wall deformities, or restrictive thoracic anatomy. Incorporating simple anthropometric and imaging-derived thoracic measurements into the interpretation of strain patterns may therefore improve diagnostic specificity.

Collectively, these findings emphasize that relative apical sparing represents a functional imaging phenotype influenced by myocardial tissue characteristics, loading conditions, ventricular geometry, and extracardiac mechanical constraints. Recognition of these alternative mechanisms is essential to avoid diagnostic oversimplification and reinforces the need for a comprehensive multiparametric evaluation when apical sparing is identified in clinical practice.

### 4.4. Clinical Implications

The present findings have important implications for the clinical application of relative apical sparing in daily practice. Although cardiac amyloidosis consistently exhibits the highest magnitude of apical sparing, the systematic review demonstrates substantial overlap in RASP values across multiple disease entities. This confirms that apical sparing should not be regarded as a standalone diagnostic criterion but rather as a supportive imaging marker that must be interpreted in conjunction with clinical presentation, ventricular morphology, and complementary imaging findings. In patients with hypertrophic phenotypes, pressure-overload states, or metabolic cardiomyopathies, reliance on relative apical sparing pattern alone may lead to false-positive interpretations and unnecessary downstream testing. Clinicians should also remain aware of non-amyloid causes of relative apical sparing—particularly narrow thoracic configuration or MVP—which may mimic the classical amyloid deformation pattern through preferential basal strain reduction [66].

From a practical perspective, when RASP values fall within an intermediate or “gray-zone” range—especially in patients with coexisting hypertrophy, aortic stenosis, or hypertrophic cardiomyopathy—diagnostic interpretation should be guided by pre-test probability rather than absolute RASP magnitude. In such borderline scenarios, a stepwise multiparametric strategy is advisable. This includes detailed evaluation of left ventricular geometry and wall thickness distribution, assessment of diastolic indices and atrial size, integration of systemic red flags (e.g., neuropathy, bilateral carpal tunnel syndrome, unexplained hypotension or intolerance to heart failure therapies), and, when clinically appropriate, escalation to tissue characterization by CMR or radionuclide bone scintigraphy. Such an approach may reduce both overdiagnosis and delayed recognition of amyloid cardiomyopathy in phenotypically overlapping conditions.

The modality-stratified analyses further highlight important practical considerations. Although STE studies demonstrated numerically larger pooled effect sizes than CMR-FT, this difference should not be interpreted as evidence of intrinsic modality superiority. Technical factors likely contribute. STE typically benefits from higher temporal resolution and frame rates, which may enhance the detection of subtle basal-to-apical strain gradients—particularly in basal segments, where dysfunction is most pronounced in amyloidosis. In contrast, CMR-FT depends on cine image temporal resolution and often incorporates greater spatial and temporal smoothing, potentially attenuating regional strain extremes and reducing apparent RASP magnitude. Additional modality-specific differences—including endocardial border definition, handling of through-plane motion, myocardial layer selection, and post-processing algorithms—may further influence segmental deformation patterns [68].

Biological and population-related factors probably also contribute to the observed discrepancy. In the present dataset, CMR-based studies included a higher proportion of hypertrophic cardiomyopathy comparators and showed lower median left ventricular mass indices compared with STE cohorts. Differences in comparator composition, disease severity, referral patterns, and stage of remodeling may therefore have narrowed intergroup contrasts in CMR studies. Because CMR is frequently performed in more selected populations with established structural disease, deformation differences between cardiac amyloidosis and comparator groups may be inherently smaller. Consequently, the modality-specific difference in pooled effect size likely reflects the combined influence of technical measurement characteristics and underlying population heterogeneity rather than a true biological discrepancy.

An additional source of variability lies in the calculation of relative apical sparing pattern itself. Although most studies defined RASP as the ratio of mean apical longitudinal strain to the sum of basal and mid-ventricular strain values, alternative formulations—such as apical-to-basal ratios or different normalization strategies—were also reported. Differences in segmental averaging methods, inclusion of myocardial layers, endocardial tracking algorithms, temporal smoothing filters, and the use of absolute versus signed strain values may substantially influence derived RASP magnitude. This lack of methodological standardization limits direct comparability across studies and imaging platforms and likely contributes to between-study heterogeneity. Accordingly, absolute RASP thresholds should be interpreted cautiously, recognizing that measurement variability may arise from computational and platform-dependent factors in addition to biological differences. Greater harmonization of RASP definitions and reporting standards would enhance reproducibility and facilitate cross-modality validation.

The discriminatory performance of relative apical sparing pattern also varies according to the comparator population. Diagnostic separation appears strongest when cardiac amyloidosis is contrasted with hypertensive heart disease, Fabry disease, or healthy controls, and weaker in comparisons with hypertrophic cardiomyopathy or advanced cardiomyopathy phenotypes. This reinforces the importance of contextual interpretation and alignment with pre-test probability.

Finally, the consistent association between elevated relative apical sparing pattern and cardiac amyloidosis across studies supports its role as a screening and triage tool in patients with unexplained left ventricular hypertrophy or heart failure with preserved ejection fraction. Current multimodality imaging recommendations emphasize that apical sparing should be integrated with conventional echocardiographic red flags (increased wall thickness, restrictive diastolic pattern, atrial enlargement), CMR-based tissue characterization, and radionuclide scintigraphy when transthyretin amyloidosis is suspected [69]. Within such an integrated framework, relative apical sparing pattern may facilitate earlier recognition of amyloid cardiomyopathy while minimizing misclassification in patients with alternative mechanical or anatomical causes of basal strain impairment.

### 4.5. Strengths and Limitations

This study has several important strengths. To the best of our knowledge, this is the first investigation to systematically integrate descriptive pooled data and quantitative meta-analytic synthesis focused specifically on the relative apical sparing pattern across cardiac amyloidosis and its major phenocopies. The inclusion of multiple disease entities associated with apical sparing allowed a comprehensive characterization of overlap and disease-specific patterns, providing a broader clinical context than previous binary comparisons. In addition, the incorporation of both STE and CMR-FT enabled modality-specific evaluation of RASP performance, which is highly relevant for real-world clinical practice. The robustness of the findings was further supported by sensitivity analyses and by the absence of statistically significant publication bias among studies included in the quantitative synthesis.

Several limitations should also be acknowledged. First, the analysis was based on aggregated study-level data rather than individual patient-level data, which limited the ability to perform more granular adjustments for confounding factors and to explore interaction effects between clinical variables and strain-derived parameters.

Second, substantial heterogeneity was observed across included studies, reflecting differences in patient populations, disease severity, imaging protocols, software platforms, and definitions of strain-derived indices. Between-study heterogeneity was extremely high in the overall analysis and particularly within the 2D-STE subgroup, indicating marked variability in effect magnitude across studies. Although random-effects models, prediction intervals, meta-regression, and sensitivity analyses were applied to account for this variability, residual heterogeneity remains significant and limits the interpretability and generalizability of pooled effect size estimates. An additional source of heterogeneity relates to the decision to pool diverse comparator populations—such as hypertensive heart disease, aortic stenosis, hypertrophic cardiomyopathy, Fabry disease, MVP, and other cardiomyopathies—into a single non-amyloid category for the primary meta-analysis. While this strategy was adopted to ensure statistical independence and to reflect real-world diagnostic uncertainty, it inevitably aggregates disease entities with distinct pathophysiological mechanisms and variable degrees of strain heterogeneity. Consequently, the pooled effect size should be interpreted as an average contrast between cardiac amyloidosis and a heterogeneous spectrum of non-amyloid phenotypes rather than as a disease-specific discriminator applicable to any single comparator condition. This methodological choice may have amplified between-study heterogeneity and influenced the magnitude of the overall standardized mean difference.

Third, not all studies used identical RASP definitions, requiring standardization and recalculation in selected cohorts. In some studies, alternative formulations (e.g., apical-to-basal ratios or different denominators) were harmonized to a predefined RASP definition when sufficient raw data were available. Although this approach improved cross-study comparability, it may have introduced measurement variability and potential misclassification bias, particularly when original definitions were not fully equivalent.

Fourth, in several studies continuous variables were reported as medians with interquartile ranges and were converted into means and standard deviations using validated statistical methods. Because strain parameters may exhibit skewed distributions, especially in small observational cohorts, such transformations may have introduced approximation error and potentially affected pooled effect estimates.

Sixth, the number of studies included in meta-regression analyses was limited. Given the small number of studies relative to the number of potential moderators, meta-regression findings should be considered exploratory and underpowered, and the absence of statistically significant moderator effects does not exclude clinically relevant sources of heterogeneity.

Seventh, variability in strain acquisition software and vendor-specific post-processing algorithms represents an additional methodological limitation. Inter-vendor differences in STE platforms and the exclusive use of non-GE software in CMR-FT studies may have introduced systematic variability in strain quantification. This may partly explain modality-specific differences in pooled effect size and raises the possibility of vendor-related bias in comparisons between STE and CMR-derived RASP values.

Eighth, lack of universal standardization in strain measurement—including differences in frame rates, tracking algorithms, segmentation models, and post-processing settings—limits cross-platform comparability and may affect reproducibility of absolute RASP values [70,71,72,73]. Accordingly, pooled thresholds should not be interpreted as universally applicable diagnostic cut-offs.

Ninth, cardiac amyloidosis subtypes (ATTR and AL) were not consistently analyzed separately across studies. Although amyloid subtype distribution was explored at the study level, a dedicated quantitative comparison between ATTR and AL amyloidosis was not feasible due to insufficient stratified data. Therefore, potential subtype-specific differences in relative apical sparing remain incompletely characterized.

Finally, publication bias was assessed only among studies included in the meta-analysis, and although no significant asymmetry was detected, the possibility of selective reporting cannot be entirely excluded.

Despite these limitations, the consistency of the observed direction of effect across modalities and comparator groups supports the robustness of the main conclusions and highlights the clinical relevance of relative apical sparing pattern as a quantitative imaging marker when interpreted within an appropriate diagnostic framework.

Future research should aim to address the methodological and clinical gaps identified in the present synthesis. In particular, large prospective multicenter studies with standardized strain acquisition protocols, harmonized post-processing algorithms, and uniform RASP definitions are needed to reduce inter-vendor variability and improve cross-platform comparability. Direct head-to-head comparisons between STE and CMR-FT within the same patient populations would further clarify modality-specific performance and reproducibility. In addition, future investigations should move beyond isolated strain metrics and evaluate the incremental diagnostic and prognostic value of RASP when integrated with complementary imaging markers—such as extracellular volume fraction, late gadolinium enhancement patterns, and radionuclide uptake grading—as well as clinical and laboratory biomarkers including natriuretic peptides, cardiac troponins, and systemic amyloid-related features. The development of multiparametric risk models and machine-learning approaches incorporating deformation indices alongside structural and biochemical markers may ultimately enhance diagnostic precision and allow more personalized stratification of patients with suspected cardiac amyloidosis.

## 5. Conclusions

Relative apical sparing pattern represents a reproducible myocardial deformation pattern that is observed across multiple cardiac conditions and is not exclusive to cardiac amyloidosis. However, cardiac amyloidosis consistently exhibits the highest magnitude of apical sparing compared with alternative causes, as confirmed by both systematic descriptive evidence and quantitative meta-analysis. Important modality-dependent differences were identified, with stronger discrimination observed using STE than CMR-FT, underscoring the need for technique-specific interpretation. These findings support the use of relative apical sparing pattern as a valuable adjunctive imaging marker for the identification of cardiac amyloidosis, while emphasizing that it should be interpreted within a comprehensive multiparametric diagnostic framework rather than as a standalone criterion.

## Figures and Tables

**Table 1:** Summary of Study Design, Imaging Methods, Population Characteristics, and Mean Relative Apical Sparing Values Across Included Studies [17,18,19,20,21,22,23,24,25,26,27,28,29,30,31,32,33,34,35,36,37,38,39,40,41,42,43,44,45].

Study Name	Design	Method	Software	Size (n)	Mean Age (% Males)	Mean RASP
Lee G.Y. (2015), S. Korea [17]	Retrosp. Multic.	2D-STE	GE	cardiac amyloidosis = 47 HHD = 72	cardiac amyloidosis = 60 (59.6) HHD = 67.5 (59.7)	cardiac amyloidosis = 0.73 HHD = 0.57
Carstersen H.G. (2016), Denmark [18]	Prosp. Multic.	2D-STE	GE	AS = 104	AS = 72 (71)	AS = 0.54
Bhatti S. (2016), USA [19]	Retrosp. Monoc.	CMR-FT	TomTec Imaging system	cardiac amyloidosis = 22 Non-amyloid group = 21	cardiac amyloidosis = 60 (74) Non-amyloid group = 61 (59)	cardiac amyloidosis = 0.76 Non-amyloid group = 0.60
Lo Q. (2016), Australia [20]	Retrosp. Multic.	2D-STE	Siemens	cardiac amyloidosis = 46 HHD = 46 Healthy = 46	cardiac amyloidosis = 60 (63) HHD = 59 Healthy = 59	cardiac amyloidosis = 0.77 HHD = 0.46 Healthy = 0.46
Williams L.K. (2017), Canada [21]	Retrosp. Monoc.	CMR-FT	Siemens	cardiac amyloidosis = 45 HCM = 19 AFD = 19	cardiac amyloidosis = 66.6 (62) HCM = 57 (74) AFD = 49.3 (68)	cardiac amyloidosis = 1.00 HCM = 0.84 AFD = 0.79
Esposito R. (2019), Italy [22]	Prosp. Monoc.	2D-STE	GE	Fabry = 23 Healthy = 23	Fabry = 37.5 (76.9) Healthy = 37.1 (76.9)	Fabry = 0.69 Healthy = 0.62
Zuo H. (2020), China [23]	Prosp. Monoc	2D-STE	GE	NIDCM = 43 IDCM = 41	NIDCM = 53.1 (76.7) IDCM = 58.9 (80.5)	NIDCM = 0.88 IDCM = 0.56
Saito M. (2020), Japan [24]	Retrosp. Multic.	2D-STE	GE	AS = 156	AS = 84 (33)	AS = 0.90
Reant P. (2020), France [25]	Prosp. Monoc.	2D-STE	GE	Fabry = 35 Healthy = 20	Fabry = 44.3 (40) Healthy = 46 (55)	Fabry = 0.72 Healthy = 0.63
Pedersen A.L.D. (2020), Denmark [26]	Retrosp. Monoc.	2D-STE	GE	AS = 499	AS = 79.8 (47)	AS = 0.84
Sonaglioni A. (2021), Italy [27]	Prosp. Monoc.	2D-STE	Philips	MVP = 60 Healthy = 60	MVP = 50.1 (53.4) Healthy = 50.6 (50)	MVP = 0.68 Healthy = 0.57
Ferreira V.V. (2021), Portugal [28]	Retrosp. Monoc.	2D-STE	GE	AS = 89	AS = 82.1 (43.8)	AS = 0.78
Robin G. (2021), France [29]	Prosp. Monoc.	2D-STE	GE	AS = 50	AS = 79 (54)	AS = 1.3
Nakao Y. (2021), Japan [30]	Retrosp. Multic.	2D-STE	GE	cardiac amyloidosis = 54 HHD = 241	cardiac amyloidosis = 75 (87) HHD = 65 (60)	cardiac amyloidosis = 1.02 HHD = 0.63
Yang C.H. (2022), Japan [31]	Retrosp. Multic.	3D-STE	Philips	AS = 230	AS = 77.9 (47)	AS = 0.76
Dohy Z. (2022), Hungary [32]	Retrosp. Monoc.	CMR-FT	Medis Medical Imaging	cardiac amyloidosis = 35 HHD = 70 HCM = 330	cardiac amyloidosis = 64.1 (64.3) HHD = 59.7 (50) HCM = 46.6 (61.5)	cardiac amyloidosis = 1.77 HHD = 1.00 HCM = 1.53
Bogunovic N. (2022), Germany [33]	Retrosp. Monoc.	2D-STE	GE	cardiac amyloidosis = 59 LVNC = 30 Healthy = 150	cardiac amyloidosis = 72.5 (77.9) LVNC = 49.4 (56.6) Healthy = 33.8 (50)	cardiac amyloidosis = 1.06 LVNC = 0.73 Healthy = 0.66
Cuddy S.A. (2022), USA [34]	Retrosp. Multic.	2D-STE	Philips	cardiac amyloidosis = 324 Non-amyloid group = 274	cardiac amyloidosis = 76 (87.1) Non-amyloid group = 68.5 (67.6)	cardiac amyloidosis = 0.90 Non-amyloid group = 0.60
Wang F. (2023), China [35]	Retrosp. Monoc.	CMR-FT	Circle Cardiovasc. Imaging Inc.	cardiac amyloidosis = 36 HCM = 37 Healthy = 36	cardiac amyloidosis = 58 (77.8) HCM = 55 (72.9) Healthy = 55 (63.9)	cardiac amyloidosis = 0.62 HCM = 0.37 Healthy = 0.41
Ferkh A. (2023), Australia [36]	Retrosp. Multic.	2D-STE	GE	cardiac amyloidosis = 120 HHD = 58 AFD = 31	cardiac amyloidosis = 68.5 (79.2) HHD = 70.3 (72.4) AFD = 46.5 (64.5)	cardiac amyloidosis = 0.85 HHD = 0.64 AFD = 0.64
Abecasis J. (2023), Portugal [37]	Prosp. Monoc.	2D-STE	GE	AS = 150	AS = 72.7 (49.3)	AS = 1.32
Singh G.K. (2023), The Netherlands [38]	Retrosp. Multic.	FT-CT	Medis Medical Imaging	AS = 156	AS = 80 (53)	AS = 1.6
Wali E. (2023), USA [39]	Retrosp. Monoc.	2D-STE	Philips	cardiac amyloidosis = 9	cardiac amyloidosis = 63 (60)	cardiac amyloidosis = 1.2
Steen H. (2024), Germany [40]	Prosp. Monoc.	CMR-FT	MyoStrain software	cardiac amyloidosis = 42 HCM = 57	cardiac amyloidosis = 73.7 (90.5) HCM = 57 (70.2)	cardiac amyloidosis = 1.2 HCM = 1.01
Cotella J. (2024), USA [41]	Retrosp. Multic.	2D-STE	GE	cardiac amyloidosis = 544 HHD = 200 Healthy = 174	cardiac amyloidosis = 73 (76.7) HHD = 74 (57) Healthy = NS	cardiac amyloidosis = 2.4 HHD = 1.6 Healthy = 1.2
Steudel T. (2024), Germany [42]	Retrosp. Monoc.	2D-STE	GE	cardiac amyloidosis = 76 AFD = 40	cardiac amyloidosis = 76 (80) AFD = 53 (48)	cardiac amyloidosis = 1.12 AFD = 0.62
Ferris T. (2024), USA [43]	Retrosp. Monoc.	2D-STE	GE	cardiac amyloidosis = 25 ESRD = 19	cardiac amyloidosis = 74.6 (80) ESRD = 48.4 (79)	cardiac amyloidosis = 1.46 ESRD = 1.42
Yang Z.X. (2025), China [44]	Retrosp. Monoc.	2D-STE	Philips	cardiac amyloidosis = 38 HCM = 20 Healthy = 16	cardiac amyloidosis = 64 (65.8) HCM = 60.8 (55) Healthy = 59.7 (50)	cardiac amyloidosis = 1.1 HCM = 0.8 Healthy = 0.6
Liu D. (2025), Germany [45]	Retrosp. Monoc.	2D-STE	GE	AS = 598	AS = 81.7 (48.7)	AS = 0.85

Values are presented as median. 2D, two-dimensional; 3D, three-dimensional; AFD, Anderson–Fabry disease; AS, aortic stenosis; cardiac amyloidosis, cardiac amyloidosis; CMR-FT, cardiac magnetic resonance feature tracking; ESRD, end-stage renal disease; FT-CT, feature-tracking computed tomography; GE, General Electric; HCM, hypertrophic cardiomyopathy; HHD, hypertensive heart disease; IDCM, ischemic dilated cardiomyopathy; LVNC, left ventricular non-compaction; Monoc., monocentric; Multic., multicentric; MVP, mitral valve prolapse; NIDCM, non-ischemic dilated cardiomyopathy; NS, not specified; Prosp., prospective; RASP, relative apical sparing pattern; Retrosp., retrospective; STE, speckle-tracking echocardiography.

**Table 2:** Pooled Baseline Clinical, Hemodynamic, Echocardiographic, and Strain Characteristics Across Study Groups.

Variable	Cardiac Amyloidosis	AS	HCM	HHD	Fabry	Other Cardiomyo.	Healthy
Age	67.5 (62.2–73.9)	79.8 (77.9–81.7)	57.0 (54.4–58.0)	66.2 (61.0–69.6)	45.4 (42.6–47.2)	49.7 (49.1–50.8)	50.6 (41.5–57.0)
Male sex	77.2 (64.0–80.0)	48.7 (47.0–53.0)	65.8 (59.9–71.1)	59.7 (57.0–60.0)	66.2 (58.4–70.2)	66.6 (55.8–77.3)	52.5 (50.0–61.7)
BMI	24.2 (23.2–25.0)	27.0 (26.8–27.1)	28.8 (28.8–28.8)	27.5 (25.8–29.3)	25.5 (25.3–25.6)	NS	23.3 (23.2–23.5)
BSA	1.90 (1.86–1.95)	1.65 (1.49–1.81)	1.94 (1.88–1.96)	1.94 (1.94–1.96)	1.81 (1.75–1.86)	1.81 (1.81–1.88)	1.83 (1.70–1.87)
SBP	112.9 (109.5–119.9)	137.0 (136.5–138.6)	130.3 (130.3–130.3)	136.5 (131.0–141.5)	123.9 (123.5–124.4)	140.2 (134.9–145.6)	120.5 (119.5–123.6)
DBP	70.0 (68.5–71.7)	71.4 (69.8–74.5)	78.8 (78.8–78.8)	76.1 (71.2–80.5)	76.0 (75.7–76.2)	82.6 (81.3–84.0)	79.1 (75.1–83.1)
HR	74.0 (72.5–75.5)	NS	65.0 (65.0–65.0)	70.0 (67.9–72.4)	65.0 (64.7–67.6)	77.0 (75.8–78.3)	68.8 (66.6–71.1)
IVS	14.9 (13.7–16.6)	13.0 (11.7–14.0)	16.30 (14.9–17.7)	11.8 (11.8–12.0)	11.6 (10.5–12.4)	12.90 (11.4–14.3)	8.7 (8.6–8.8)
PW	14.5 (13.2–16.2)	11.6 (10.9–12.7)	11.7 (11.7–11.7)	11.4 (11.2–11.6)	7.7 (7.7–7.7)	12.9 (11.4–14.3)	8.9 (7.8–9.1)
RWT	0.68 (0.64–0.75)	0.53 (0.51–0.56)	0.60 (0.60–0.60)	0.53 (0.53–0.56)	0.42 (0.37–0.47)	0.32 (0.32–0.32)	0.35 (0.33–0.38)
LVMi	112.2 (90.9–128.1)	110.4 (95.2–134.2)	89.2 (82.1–89.3)	104.0 (91.5–126.5)	NS	NS	64.0 (43.2–78.9)
LVEDD	43.3 (41.67–45.00)	48.9 (47.5–50.4)	46.5 (46.5–46.5)	45.6 (43.3–47.3)	48.2 (47.7–48.8)	62.0 (53.3–64.0)	48.6 (48.3–49.3)
LVEDVi	72.0 (43.60–80.90)	64.6 (53.0–79.4)	NS	NS	48.5 (48.4–48.7)	60.0 (54.8–69.5)	NS
LVEF	51.0 (49.80–54.45)	54.9 (52.8–57.0)	60.8 (59.0–62.1)	61.0 (54.7–61.3)	64.6 (62.8–65.1)	42.5 (32.7–54.2)	64.1 (63.1–64.2)
E/A	1.75 (1.30–1.90)	0.92 (0.80–1.11)	1.10 (1.10–1.10)	0.97 (0.83–1.10)	1.25 (1.25–1.26)	1.38 (1.09–1.49)	1.20 (1.10–1.29)
E/e’	18.3 (17.5–19.9)	19.0 (17.3–20.2)	13.5 (13.5–13.5)	14.0 (11.7–15.1)	8.9 (8.2–9.6)	17.5 (13.6–21.3)	7.6 (6.8–8.3)
LAVi	46.5 (45.0–48.7)	44.8 (43.9–46.8)	NS	41.1 (38.6–44.1)	35.6 (32.1–35.6)	52.0 (41.5–67.4)	24.9 (22.5–32.2)
TAPSE	17.4 (16.1–18.1)	19.3 (18.9–19.8)	NS	16.9 (16.9–16.9)	22.5 (22.5–22.5)	22.3 (22.3–22.3)	23.0 (23.0–23.0)
LV-GLS	11.8 (10.8–13.9)	12.9 (12.1–14.0)	16.7 (15.3–19.4)	15.2 (14.2–17.9)	19.5 (17.2–21.7)	11.1 (9.6–13.7)	21.1 (18.5–22.0)
Mean basal LS	9.5 (7.0–12.2)	9.0 (6.8–10.9)	16.3 (15.1–18.0)	16.4 (13.6–16.5)	16.1 (14.2–18.5)	6.8 (6.5–10.4)	19.2 (18.2–20.6)
Mean mid LS	11.1 (9.7–13.7)	12.5 (10.9–12.9)	16.8 (15.5–19.0)	14.8 (14.3–18.4)	18.4 (16.2–20.6)	9.8 (9.2–13.8)	20.4 (16.8–21.2)
Mean apical LS	17.4 (14.1–20.7)	18.2 (17.0–19.2)	23.7 (19.0–28.1)	20.3 (15.9–22.2)	24.1 (21.2–27.6)	19.3 (15.5–20.4)	24.6 (17.9–25.4)
Diuretics	NS	56.5 (54.0–59.0)	NS	NS	NS	81.4 (81.4–81.4)	NS
ACEi/ARBs	NS	50.0 (42.0–58.0)	NS	NS	3.0 (3.0–3.0)	55.1 (15.0–95.3)	11.6 (11.6–11.6)
Antiplatelets	NS	55.5 (53.0–58.0)	NS	NS	NS	31.0 (13.3–48.8)	10.0 (10.0–10.0)
Anticoagulants	NS	34.5 (33.0–36.0)	NS	NS	NS	NS	NS
BB	NS	59.5 (52.0–67.0)	NS	NS	NS	51.3 (16.6–86)	13.3 (13.3–13.3)
Amiodarone	NS	14.0 (14.0–14.0)	NS	NS	NS	NS	NS
CCB	NS	14.0 (14.0–14.0)	NS	NS	NS	9.3 (9.3–9.3)	NS
Statins	NS	68.0 (68.0–68.0)	NS	NS	NS	42.5 (20.0–65.1)	16.6 (16.6–16.6)

Data are presented as study-level medians with associated ranges. ACEi, angiotensin-converting enzyme inhibitor; ARB, angiotensin receptor blocker; AS, aortic stenosis; BB, beta-blocker; BMI, body mass index; BSA, body surface area; cardiac amyloidosis, cardiac amyloidosis; cardiomyo., cardiomyopathies; CCB, calcium channel blocker; DBP, diastolic blood pressure; E/A, early-to-late mitral inflow velocity ratio; E/e’, ratio of early mitral inflow velocity to mitral annular early diastolic velocity; Fabry, Anderson–Fabry disease; HCM, hypertrophic cardiomyopathy; HHD, hypertensive heart disease; HR, heart rate; IVS, interventricular septal thickness; LAVi, left atrial volume index; LS, longitudinal strain; LVEDD, left ventricular end-diastolic diameter; LVEDVi, left ventricular end-diastolic volume index; LVEF, left ventricular ejection fraction; LV-GLS, left ventricular global longitudinal strain; LVMi, left ventricular mass index; NS, not specified; PW, posterior wall thickness; RWT, relative wall thickness; SBP, systolic blood pressure; TAPSE, tricuspid annular plane systolic excursion.

**Table 3:** Study-Level Comparison Between 2D-STE and CMR-FT Subgroups.

Variable	2D-STE (n = 9)	CMR-FT (n = 5)	*p*-Value
*Study-Level Baseline Clinical and Echocardiographic Characteristics*			
Age (years)	72.5 (64.0–74.6)	64.1 (60.0–66.6)	0.228
BSA (m^2^)	1.84 (1.83–1.90)	1.85 (1.85–1.86)	0.687
SBP (mmHg)	114.0 (112.9–116.0)	114.0 (114.0–114.0)	0.835
HR (bpm)	75.0 (72.8–77.0)	76.0 (76.0–76.0)	0.532
LVMI (g/m^2^)	108.0 (107.0–124.0)	90.5 (88.3–91.9)	**0.003**
LVEF (%)	52.0 (50.0–55.1)	51.0 (50.4–53.8)	0.894
E/e’	18.8 (17.8–19.6)	20.0 (19.0–20.0)	0.422
Male sex (%)	77.9 (65.8–80.0)	74.0 (64.3–77.8)	0.689
Diabetes (%)	16.5 (16.0–16.5)	16.5 (16.0–17.0)	0.946
*Comparator Composition*			
Healthy controls included	4 (44%)	1 (20%)	0.58
HHD included	3 (33%)	1 (20%)	1.00
HCM included	0 (0%)	3 (60%)	**0.03**
Mixed comparators	1 (11%)	2 (40%)	0.24
*S* *train Software Platform Distribution*			
GE-based	7 (78%)	0 (0%)	**0.01**
Non-GE	2 (22%)	5 (100%)	**0.01**

Study-level baseline clinical and echocardiographic characteristics, comparator composition, and strain software platform distribution across studies using two-dimensional speckle-tracking echocardiography and cardiac magnetic resonance feature tracking. Continuous variables are presented as medians with interquartile ranges; categorical variables are reported as number of studies and percentages. Statistical comparisons between subgroups were performed using Mann–Whitney U tests for continuous variables and Fisher’s exact test for categorical variables. Statistically significant *p*-values are reported in bold type. 2D-STE, two-dimensional speckle-tracking echocardiography; BSA, body surface area; CMR-FT, cardiac magnetic resonance feature tracking; E/e’, ratio of early mitral inflow velocity to mitral annular early diastolic velocity; GE, General Electric; HCM, hypertrophic cardiomyopathy; HHD, hypertensive heart disease; HR, heart rate; LVEF, left ventricular ejection fraction; LVMI, left ventricular mass index; SBP, systolic blood pressure.

**Table 4:** Meta-Regression Assessment of Factors Affecting Effect Size Estimates.

Covariate	Coefficient	Standard Error	95% Lower	95% Upper	*p*-Value
Intercept	29.492	54.971	−78.249	137.232	0.592
Software: NonGE	−0.404	1.442	−3.231	2.422	0.779
AL-cardiac amyloidosis	−0.017	0.049	−0.113	0.079	0.728
Age (yrs)	−0.039	0.208	−0.446	0.369	0.853
% Males	−0.002	0.065	−0.129	0.126	0.979
BSA (m^2^)	−7.684	4.917	−17.322	1.953	0.118
SBP (mmHg)	−0.094	0.068	−0.227	0.040	0.168
HR (bpm)	−0.018	0.520	−1.038	1.002	0.972
% Diabetes	−0.016	0.061	−0.136	0.105	0.798
LVMi (g/m^2^)	0.046	0.033	−0.019	0.112	0.165
LVEF (%)	−0.031	0.135	−0.295	0.233	0.818
E/e’	−0.036	0.125	−0.280	0.209	0.775

Meta-regression coefficients are reported with corresponding standard errors and 95% confidence intervals. Covariates represent study-level characteristics evaluated as potential moderators of the pooled effect size. Positive coefficients indicate an increase in the effect estimate, whereas negative coefficients indicate a decrease. AL-cardiac amyloidosis, amyloid light-chain cardiac amyloidosis; BSA, body surface area; E/e’, ratio of early mitral inflow velocity to mitral annular early diastolic velocity; GE, General Electric; HR, heart rate; LVEF, left ventricular ejection fraction; LVMi, left ventricular mass index; SBP, systolic blood pressure.

## Data Availability

Data extracted from included studies will be publicly available on Zenodo (https://zenodo.org).

## Supplementary Materials

The following supporting information can be downloaded at: https://www.mdpi.com/article/10.3390/jcm15051685/s1, S1: PRISMA 2020 checklist; S2: The full INPLASY record (https://inplasy.com/inplasy-2026-1-0080/, accessed on 23 January 2026); S3: NIH Quality Assessment of included studies [17,19,20,21,30,32,33,35,36,40,41,42,43,44]; S4: Traffic-light plot of risk-of-bias assessment across included studies [17,19,20,21,30,32,33,35,36,40,41,42,43,44]; S5: Overview of risk-of-bias judgments across included studies [17,19,20,21,30,32,33,35,36,40,41,42,43,44].
