# Supplementary material for: Diagnostic Performance of Relative Apical Sparing Across Cardiac Diseases: A Multimodality Systematic Review and Meta-Analysis
Source: J Clin Med. 2026 Feb 24;15(5):1685. doi: 10.3390/jcm15051685 (PMC12986376; doi:10.3390/jcm15051685)
Supplement: Supplementary file 1 [file jcm-15-01685-s001.zip › Supplementary Materials S3.pdf]

Supplementary Materials S3. Risk of Bias Assessment (NIH Quality Assessment Tool for Case–Control Studies)

Responses: YES = criterion met; NO = criterion not met; NR = not reported/cannot determine; NA = not applicable.

Study	Q1	Q2	Q3	Q4	Q5	Q6	Q7	Q8	Q9	Q10	Q11	Q12	Quality score (0–12)	Overall quality (Good/Fair/Poor)
Lee G.Y. 2015	YES	YES	NO	YES	YES	YES	NA	YES	NO	YES	YES	NR	8	Fair
Bhatti 2016	YES	YES	NO	YES	YES	YES	NA	YES	NO	YES	YES	NR	8	Fair
Lo Q 2016	YES	YES	NO	YES	YES	YES	NR	YES	NO	YES	NR	NR	7	Fair
Williams L.K. 2017	YES	YES	NO	YES	YES	YES	YES	YES	NO	YES	YES	YES	10	Good
Nakao Y. 2021	YES	YES	YES	YES	YES	YES	NA	YES	NO	YES	YES	YES	10	Good
Dohy Z. 2022	YES	YES	NO	YES	YES	YES	NR	YES	NO	YES	NR	NR	7	Fair
Bogunovic N. 2022	YES	YES	NO	YES	YES	YES	NR	YES	NO	YES	NR	NR	7	Fair
Wang F. 2023	YES	YES	NO	YES	YES	YES	NR	YES	NO	YES	NR	NR	7	Fair
Ferkh A. 2023	YES	YES	NO	YES	YES	YES	NA	YES	NO	YES	NR	YES	8	Fair
Steen H. 2024	YES	YES	NO	YES	YES	YES	NR	YES	NO	YES	NR	YES	8	Fair
Steudel T. 2024	YES	YES	NO	YES	YES	YES	NR	YES	NO	YES	NR	YES	8	Fair
Cotella J. 2024	YES	YES	NO	YES	YES	YES	NR	YES	NO	YES	NR	NR	7	Fair
Ferris T. 2024	YES	YES	NO	YES	YES	YES	NR	YES	NO	YES	NR	NR	7	Fair
Yang Z.X. 2025	YES	YES	NO	YES	YES	YES	NR	YES	NO	YES	NR	NR	7	Fair
