# Supplementary material for: Diagnostic Performance of Relative Apical Sparing Across Cardiac Diseases: A Multimodality Systematic Review and Meta-Analysis
Source: J Clin Med. 2026 Feb 24;15(5):1685. doi: 10.3390/jcm15051685 (PMC12986376; doi:10.3390/jcm15051685)
Supplement: Supplementary file 1 [file jcm-15-01685-s001.zip › Supplementary Materials S2.pdf]

International Platform of Registered Systematic Review and Meta-analysis Protocols

INPLASY

INPLASY202610080
doi: 10.37766/inplasy2026.1.0080
Received: 23 January 2026
Published: 23 January 2026

Corresponding author:
Andrea Sonaglioni

sonaglioniandrea@gmail.com

Author Affiliation:
MultiMedica.

Diagnostic Performance of Relative Apical Sparing Across Cardiac Diseases: A Multimodality Systematic Review and Meta-Analysis

Sonaglioni, A; Gramaglia, GF; Nicolosi, GL; Baravelli, M; Lombardo, M.

ADMINISTRATIVE INFORMATION

Support - No funding.

Review Stage at time of this submission - Completed but not published.

Conflicts of interest - None declared.

INPLASY registration number: INPLASY202610080

Amendments - This protocol was registered with the International Platform of Registered Systematic Review and Meta-Analysis Protocols (INPLASY) on 23 January 2026 and was last updated on 23 January 2026.

INTRODUCTION

Review question / Objective The aims of the present study were twofold. First, we performed a systematic review of all available studies reporting the relative apical sparing pattern (RASP) across cardiac amyloidosis and other disease entities associated with apical sparing patterns, including aortic stenosis, hypertrophic cardiomyopathy, hypertensive heart disease, Fabry disease, healthy controls, and other cardiomyopathies. Second, we conducted a meta-analysis comparing cardiac amyloidosis with non-amyloid conditions to quantitatively assess the magnitude of RASP differences and to evaluate potential modality-specific effects between speckle-tracking echocardiography and cardiac magnetic resonance feature tracking.
By integrating descriptive pooled data with quantitative meta-analytic synthesis, this study aims to clarify the clinical meaning of relative apical sparing, to better define its disease specificity, and to provide a comprehensive framework for the interpretation of RASP in daily clinical practice.

Rationale Despite the expanding literature on relative apical sparing, several important gaps remain. First, most available studies focus on binary comparisons between cardiac amyloidosis and a single control group, limiting the ability to contextualize RASP values across a broad spectrum of cardiac diseases. Second, reported RASP values vary substantially across studies, imaging platforms, and post-processing software, making it difficult to establish reference ranges and disease-specific distributions. Third, although multiple meta-analyses have addressed global longitudinal strain in amyloidosis, a comprehensive quantitative synthesis specifically focused on the relative apical sparing ratio across imaging modalities is currently lacking.
Furthermore, with the increasing adoption of cardiac magnetic resonance feature tracking (CMR-FT) as an alternative to speckle-tracking

Sonaglioni et al. INPLASY protocol 202610080. doi:10.37766/inplasy2026.1.0080 Downloaded from https://inplasy.com/inplasy-2026-1-0080/

echocardiography, the comparative performance of RASP derived from different imaging techniques has not been systematically evaluated. Whether the magnitude of apical sparing differs between echocardiographic and CMR-based strain analysis and how this affects diagnostic discrimination remains incompletely understood.

Condition being studied Relative apical sparing of longitudinal strain has emerged as one of the most widely recognized imaging signatures of cardiac amyloidosis. Since its first description, the preservation of apical deformation in the presence of marked basal and mid-ventricular dysfunction has been increasingly used as a non-invasive marker to support the diagnosis of amyloid cardiomyopathy and to differentiate it from other causes of left ventricular hypertrophy and heart failure.

The relative apical sparing pattern is commonly quantified using ratio-based indices derived from regional longitudinal strain, most frequently expressed as the ratio between apical strain and the sum of basal and mid-ventricular strain values. This relative apical sparing ratio (RASP) captures the characteristic base-to-apex gradient of myocardial dysfunction observed in cardiac amyloidosis and has demonstrated good diagnostic performance in several single-center and multicenter studies. As a result, RASP has been incorporated into clinical workflows and is increasingly applied in routine echocardiographic and cardiac magnetic resonance strain analysis.

However, growing evidence suggests that apical sparing is not a pathognomonic feature of cardiac amyloidosis. Similar regional deformation patterns have been reported in other clinical conditions, including pressure overload states such as aortic stenosis, hypertrophic phenotypes such as hypertrophic cardiomyopathy and Fabry disease, and selected forms of hypertensive heart disease and advanced cardiomyopathies. These observations raise important clinical questions regarding the specificity of the apical sparing pattern and the magnitude of overlap between cardiac amyloidosis and its phenocopies.

METHODS

Search strategy A comprehensive literature search was independently conducted by two investigators to identify all relevant studies reporting the RASP or equivalent regional strain-based indices in patients with cardiac amyloidosis and comparator populations.

The electronic databases PubMed, Scopus, and EMBASE were systematically searched from database inception to December 2025. The search strategy combined controlled vocabulary terms and free-text keywords related to cardiac amyloidosis, myocardial strain analysis, and apical sparing patterns.

The following search terms and Boolean operators were used in various combinations: “cardiac amyloidosis” OR “amyloid cardiomyopathy” OR “ATTR amyloidosis” OR “AL amyloidosis” AND “apical sparing” OR “relative apical sparing pattern” OR “RASP” OR “regional longitudinal strain” AND “echocardiography” OR “speckle tracking” OR “strain imaging” OR “cardiac magnetic resonance” OR “feature tracking” OR “CMR strain”.

No restrictions were applied regarding language, publication year, or study design. In addition, the reference lists of all included articles and relevant review papers were manually screened to identify potentially eligible studies not captured by the electronic search.

Any discrepancies between reviewers during the screening and selection process were resolved by discussion and consensus. When agreement could not be reached, a third investigator was consulted for adjudication.

Participant or population Patients with cardiac amyloidosis and/or other disease entities associated with relative apical sparing patterns, including aortic stenosis, hypertrophic cardiomyopathy, hypertensive heart disease, Fabry disease, healthy controls, and other cardiomyopathies, who underwent a quantitative assessment of myocardial deformation with reporting of regional longitudinal strain values or direct calculation of the RASP, obtained using speckle-tracking echocardiography or cardiac magnetic resonance feature tracking.

Intervention N/A.

Comparator For quantitative synthesis, the primary meta-analysis compared cardiac amyloidosis groups versus non-cardiac amyloidosis groups, including all alternative disease entities associated with relative apical sparing patterns such as aortic stenosis, hypertrophic cardiomyopathy, hypertensive heart disease, Fabry disease, healthy controls, and other cardiomyopathies.

Study designs to be included Studies were considered eligible for inclusion if they had an observational design, including cross-sectional, case–control, or cohort studies, and evaluated patients with cardiac amyloidosis and/or other disease entities associated with relative apical sparing patterns, including aortic stenosis,

Sonaglioni et al. INPLASY protocol 202610080. doi:10.37766/inplasy2026.1.0080 Downloaded from https://inplasy.com/inplasy-2026-1-0080/

hypertrophic cardiomyopathy, hypertensive heart disease, Fabry disease, healthy controls, and other cardiomyopathies.

Eligibility criteria Studies were considered eligible for inclusion if they had an observational design, including cross-sectional, case–control, or cohort studies, and evaluated patients with cardiac amyloidosis and/or other disease entities associated with relative apical sparing patterns, including aortic stenosis, hypertrophic cardiomyopathy, hypertensive heart disease, Fabry disease, healthy controls, and other cardiomyopathies. Eligible studies were required to provide a quantitative assessment of myocardial deformation with reporting of regional longitudinal strain values or direct calculation of the RASP, obtained using speckle-tracking echocardiography or cardiac magnetic resonance feature tracking. Furthermore, studies had to report clearly defined diagnostic criteria for cardiac amyloidosis and comparator conditions based on histology, imaging findings, or guideline-recommended clinical algorithms, and to provide extractable quantitative data on RASP or on apical, mid-ventricular, and basal longitudinal strain values allowing standardized computation of the RASP, reported as mean ± standard deviation, median with interquartile range, or in a format suitable for statistical transformation. Preference was given to studies reporting baseline clinical and echocardiographic variables, including demographic characteristics, comorbidities, and pharmacological therapy.
Studies were excluded if they did not include strain-based regional deformation analysis or if insufficient information was available to derive the RASP, if they enrolled mixed or poorly characterized populations without a clear distinction between cardiac amyloidosis and comparator disease groups, if they used experimental or non-clinical imaging protocols without standardized strain quantification, if quantitative data were insufficient to allow effect size calculation or pooled analysis, or if they were non-original publications such as editorials, conference abstracts, letters, case reports, narrative reviews, expert opinions, or guidelines.

Information sources The electronic databases PubMed, Scopus, and EMBASE were systematically searched from database inception to December 2025.

Main outcome(s) By integrating descriptive pooled data with quantitative meta-analytic synthesis, this study aims to clarify the clinical meaning of relative apical sparing, to better define its disease specificity, and to provide a comprehensive framework for the interpretation of RASP in daily clinical practice.

Quality assessment / Risk of bias analysis The methodological quality and risk of bias of the included studies were independently evaluated by two investigators using the National Institutes of Health (NIH) Quality Assessment Tool for Case–Control Studies. This tool assesses multiple methodological domains related to study design, population selection, case and control definition, exposure assessment, outcome ascertainment, blinding procedures, and control of confounding factors. Each study was evaluated across all predefined criteria and classified according to NIH guidance. Inter-rater agreement between reviewers was quantified using Cohen's kappa coefficient. Any disagreement regarding individual domain ratings or overall quality classification was resolved through discussion and re-evaluation of the original manuscripts until consensus was achieved. Publication bias was assessed only among studies included in the quantitative meta-analysis, as funnel plot-based methods require comparable effect size estimates.

Strategy of data synthesis Two investigators independently screened all retrieved records by title and abstract, followed by full-text evaluation of potentially eligible studies according to the predefined inclusion and exclusion criteria. Disagreements regarding study eligibility were resolved by discussion and consensus, and when consensus could not be achieved, a third reviewer was consulted for adjudication. Data extraction was independently performed by the same investigators using a standardized and pre-specified data collection form. Extracted variables included study characteristics such as first author, year of publication, country, study design, imaging modality, and sample size for each study group; demographic and anthropometric parameters including age, sex distribution, body mass index, and body surface area; clinical comorbidities and cardiovascular risk factors such as hypertension, diabetes mellitus, dyslipidemia, smoking history, atrial fibrillation, and history of coronary artery disease; laboratory parameters when available, including hemoglobin, renal function indices, natriuretic peptides, and serum biomarkers; echocardiographic and structural parameters including left ventricular wall thickness, relative wall thickness, left ventricular mass index, chamber dimensions, left ventricular ejection fraction, stroke volume index, indices of diastolic function, left atrial volume index, pulmonary artery systolic pressure, right ventricular systolic function

Sonaglioni et al. INPLASY protocol 202610080. doi:10.37766/inplasy2026.1.0080 Downloaded from https://inplasy.com/inplasy-2026-1-0080/

parameters, and presence of pericardial effusion; strain-derived parameters including global longitudinal strain, mean basal, mid-ventricular, and apical longitudinal strain values, directly reported RASP values, and study-specific diagnostic cut-offs when available; baseline pharmacological therapy including antiplatelet agents, anticoagulants, beta-blockers, angiotensin-converting enzyme inhibitors, angiotensin receptor blockers, calcium channel blockers, diuretics, statins, antiarrhythmic drugs, and disease-specific therapies; and summary statistics including means with standard deviations or medians with interquartile ranges, together with corresponding confidence intervals and p-values. When numerical data were reported exclusively in graphical format, values were extracted using WebPlotDigitizer (version 4.6), and when necessary for quantitative synthesis, medians and interquartile ranges were converted into means and standard deviations using established statistical conversion methods. All extracted data were systematically cross-checked for accuracy, and discrepancies between reviewers were resolved by re-evaluation of the original articles until agreement was reached.

Subgroup analysis 2D-STE studies vs. CMR-FT studies assessing RASP in CA and nonamyloid groups.

Sensitivity analysis Sensitivity analyses were performed when appropriate to assess the robustness of pooled estimates.

Language restriction No language restriction.

Country(ies) involved Italy.

Keywords RASP; cardiac amyloidosis; Fabry; HCM; hypertensive heart disease; mitral valve prolapse; meta-analysis.

Contributions of each author

Author 1 - Andrea Sonaglioni - Author 1 drafted the manuscript.
Email: sonaglioniandrea@gmail.com
Author 2 - Giulio Francesco Gramaglia - The authors contributed to the Search Strategy.
Email: giulio.gramaglia@unimi.it
Author 3 - Gian Luigi Nicolosi - The authors critically revised the original manuscript.
Email: gianluigi.nicolosi@gmail.com
Author 4 - Massimo Baravelli - The author read, provided feedback and approved the final manuscript.
Email: massimo.baravelli@multimedica.it
Author 5 - Michele Lombardo - The author read, provided feedback and approved the final manuscript.

Sonaglioni et al. INPLASY protocol 202610080. doi:10.37766/inplasy2026.1.0080 Downloaded from https://inplasy.com/inplasy-2026-1-0080/
