# Supplementary figures and images for: Diagnostic Performance of Relative Apical Sparing Across Cardiac Diseases: A Multimodality Systematic Review and Meta-Analysis
Source: J Clin Med. 2026 Feb 24;15(5):1685. doi: 10.3390/jcm15051685 (PMC12986376; doi:10.3390/jcm15051685)

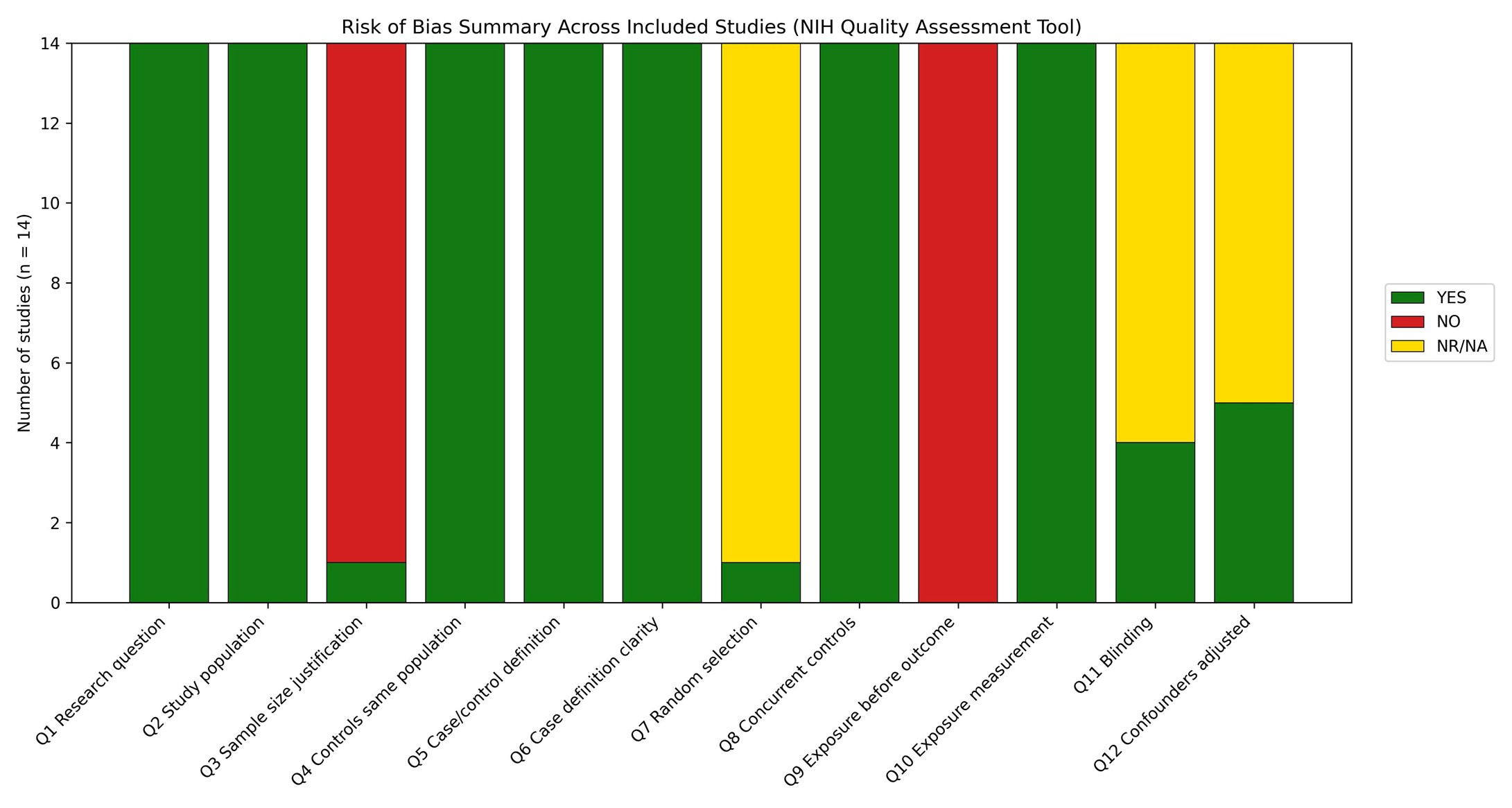

Supplement: Supplementary file 1 [file jcm-15-01685-s001.zip › Supplementary Materials S5.jpg]

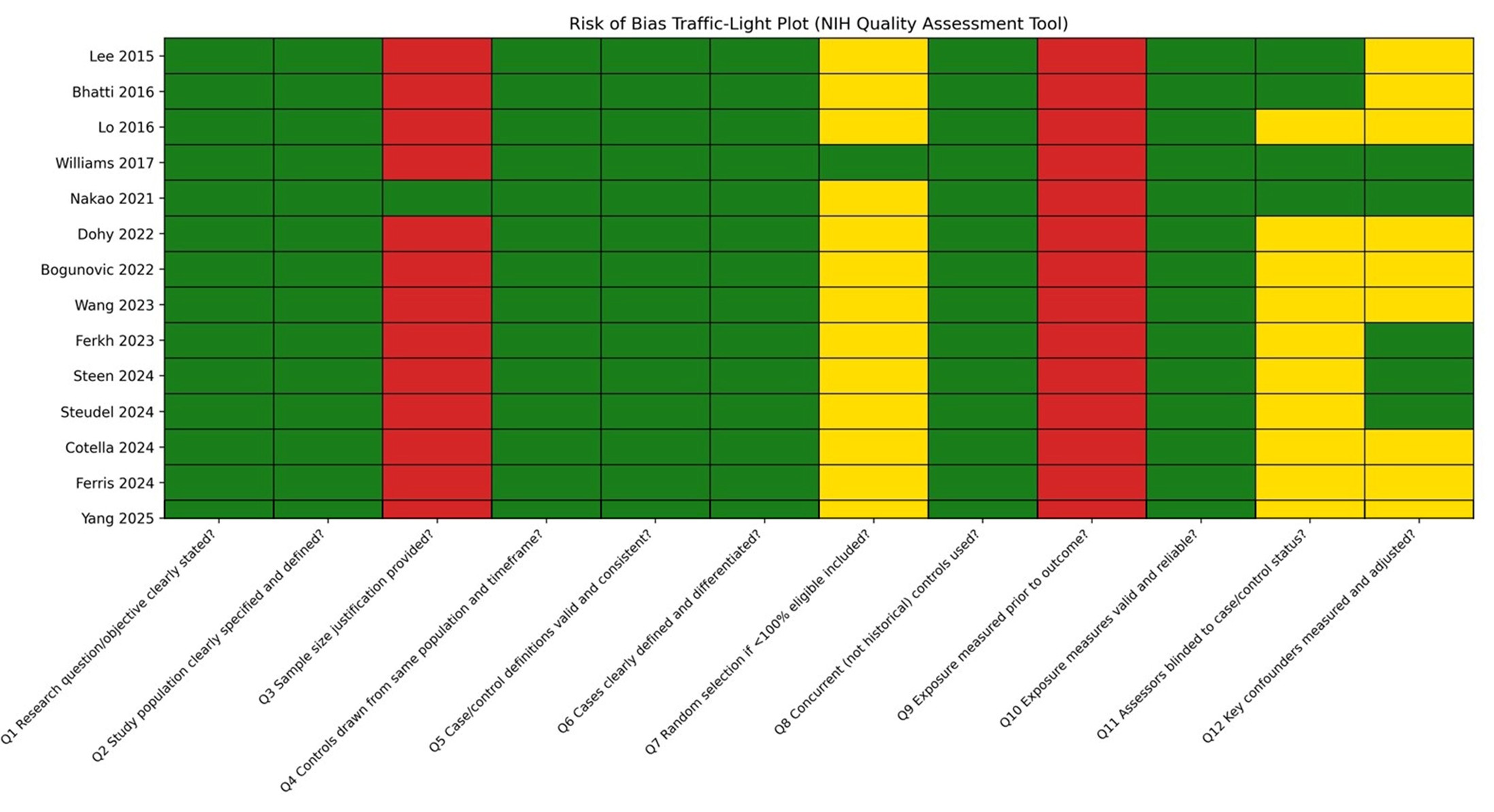

Supplement: Supplementary file 1 [file jcm-15-01685-s001.zip › Supplementary Materials S4.jpg]
